# Treating social cognition impairment with the online therapy ’SoCoBo’: A randomized controlled trial including traumatic brain injury patients

**DOI:** 10.1371/journal.pone.0294767

**Published:** 2024-01-10

**Authors:** Tobias Lohaus, Sally Reckelkamm, Patrizia Thoma

**Affiliations:** Neuropsychological Therapy Centre (NTC), Faculty of Psychology, Ruhr-University Bochum, Bochum, North Rhine-Westphalia, Germany; Jordan University of Science and Technology, JORDAN

## Abstract

**Objective:**

Acquired brain injuries (ABIs), such as traumatic brain injuries (TBIs), often entail impairments of general cognition (e.g., memory, attention or executive functions) and social cognition (e.g. emotion recognition, theory of mind [ToM], social problem-solving). The availability of fully computerized interventions targeting sociocognitive deficits specifically in neurologically impaired patients is extremely limited. Therefore, the Treatment Program for Deficits in Social Cognition and Social Competencies of the Ruhr University Bochum (SoCoBo), a fully computerized online therapy designed for ABI patients was evaluated in a randomized controlled trial involving TBI patients.

**Method:**

Sixty-four patients with TBI were randomly assigned to two groups with 43 patients fully completing either SoCoBo (*N* = 27) or a commercially available computerized program for cognitive rehabilitation (RehaCom^®^, *N* = 16). All participants underwent comprehensive pre-post online neuropsychological assessment and worked with their respective rehabilitation programs for four days a week during a scheduled period of 12 weeks.

**Results:**

After treatment, the SoCoBo group, but not the RehaCom^®^ group showed significant improvements in facial emotion recognition and self-rated empathy. Moreover, in the SoCoBo group, an increase in empathy was also associated with increased life satisfaction after treatment. There were no improvements in ToM and social problem-solving. Furthermore, general cognition did not improve in any of the groups.

**Conclusions:**

SoCoBo represents an effective new online therapy for the amelioration of deficits in key domains of social cognition. Its implementation in clinical practice will serve as a meaningful addition to the existing fully computerized approaches specifically in neurological patient groups.

## Introduction

Acquired brain injury (ABI) represents any injury or disease affecting the brain that is not a congenital or developmental disorder [1] and can be a consequence of both non-traumatic (e.g., stroke) or traumatic (traumatic brain injury, TBI) events, such as a penetrating injury to the head [2]. ABIs can entail a wide range of impairments [3, 4], persisting even years after the injury [5]. Apart from motor impairments [6] and deficits in general cognition compromising attention, memory, and executive functions [7] that have been studied extensively, it becomes increasingly evident that also a wide range of domains of social cognition and social behavior can be impaired [8–10], affecting the patients’ lives in the long term [11].

Social cognition is an umbrella term for various, partly interrelated abilities [12]: Emotion recognition denotes the ability to perceive emotional cues and to link them to stored knowledge of emotional expressions [13]. ToM is the ability to infer and understand other people’s thoughts and emotions [14]. The understanding of thoughts is often referred to as *cognitive ToM*, and the understanding of emotions as *affective ToM* [15]. The related concept of empathy comprises an individual’s ability to infer, affectively resonate with and to affectively respond to other people’s emotional states [16]. Understanding another individual’s emotional states is referred to as *cognitive empathy*, experiencing feelings of concern to another persons’ emotional state (without completely sharing this state) is called *affective empathy* [17]. A*ffective ToM* is usually considered as synonymous with *cognitive empathy*. With regard to more behavior-related domains of social cognition, for which emotion recognition and ToM can be regarded as preconditions, recognizing a social conflict and then finding an appropriate and effective strategy to overcome this social conflict is regarded as social problem-solving [18].

Several studies suggest that social cognition impairment might mediate the link between general cognition and functional outcome [19–21], although there are also contradicting findings suggesting that there is no correlation between social cognition and general cognition [22]. When it comes to the neural underpinnings of social cognition deficits, the ventromedial prefrontal cortex seems to be especially relevant [22], albeit a widespread network of fronto-temporal and subcortical areas is thought to mediate distinct components of social cognition [23]. In general, impairments in various sociocognitive domains can be linked to massive changes in social life (e.g., poorer social participation [24]) not only for the ABI patients themselves, but also for the family caregivers [25]. Moreover, social cognition impairment can be linked to difficulties in community integration [26], vocational reintegration [27] and family-related stress [28]. Furthermore, a link between depression and anxiety and social cognition has been discussed (e.g., for the social cognition domain emotion perception [9]) and both depression and sociocognitive impairment contribute to functional outcome following TBI rendering it necessary to consider depressive symptoms when treating social cognition impairment [29]. It is unfortunate that social cognition impairment has not been focussed on with regard to assessment and intervention [30–32] and it appears absolutely crucial to develop treatments for people with ABI that target deficits in this domain, also taking into account general cognitive impairment since it appears to be related to social cognition.

While numerous social cognition therapies that are delivered on-site already exist (e.g., Social Cognition and Interaction Training, SCIT [33]), in general, computerized approaches offer several advantages over non-computerized approaches, for example in that they can be carried out in flexible locations [34]. In terms of social domains, computerized approaches also yield some additional advantages, e.g., the complex social world can be deconstructed into smaller components in order to focus exclusively on details [35].

A number of fully computerized, not therapeutically assisted social cognition interventions exist for autism [36, 37] and especially–as summarized in a systematic review by Lohaus et al. [38]–for schizophrenia patients, many of which have been associated with positive effects on social cognitive outcomes, especially on the social cognition subdomain emotion recognition. However, these interventions predominantly target a single social cognition domain, although social cognition encompasses a wide variety of interrelated subdomains. There is a complete dearth of fully computerized social cognition therapies specifically developed for TBI patients. One partly, but not fully computerized social cognition intervention was introduced and well-evaluated by Westerhof-Evers et al. [39]: The Treatment for Impairments in Social Cognition and Emotion Regulation (T-ScEmo). This intervention covers different modules of social cognition (emotion recognition, ToM, and social behavior), with only the emotion recognition module being computerized. Significant effects in favor of T-ScEmo were revealed with regard to emotion recognition, ToM and social behavior. To investigate whether these results can also be replicated using a not only partially, but fully computerized approach with only minimal support provided by therapist, the Treatment Program for Deficits in Social Cognition and Social Competencies of the Ruhr University Bochum (SoCoBo) which covers similar social cognition domains compared to T-ScEmo (emotion recognition, cognitive and affective perspective taking [ToM / empathy], social problem-solving) has been developed. Just as Westerhof-Evers et al. [39] and following the theoretical categorization of sociocognitive subdomains into social perception (emotion recognition), perspective taking (ToM/empathy) and social behavior (assessing social problem solving as a proxy concept) proposed for example by Henry et al. [23], we assume that a comprehensive, multifaceted intervention, targeting several aspects of social cognition, should be more effective compared to targeting only a single domain of social cognition. The interrelated nature of the individual social cognition domains suggests that improvements in a particular domain can carry over to improvements in another domain [40].

The aim of the current study is to evaluate the effectiveness of the new SoCoBo online therapy with regard to improving sociocognitive impairment with its subdomains being regarded as the primary outcomes as they are considered the domains SoCoBo especially targets on in TBI patients. A comprehensive randomized controlled trial (RCT) study was conducted including a computerized cognitive rehabilitation control group. The reason why a computerized cognitive rehabilitation control group (RehaCom^®^, which will be described in more detail in the treatment section of this article [41]) was implemented is that the focus in this group is on the improvement of general cognitive functioning in the domains of attentional, memory and executive functioning. Thus, improvements observed following treatment with SoCoBo, as compared to RehaCom^®^, should be driven mainly by the intervention that was specifically designed to address sociocognitive functioning. Also RehaCom^®^, unlike SoCoBo, can be regarded as an already established intervention program that is much used in therapeutic practice and works in a similar internet-based manner. Ideally, the benefits of SoCoBo should also extend to mental health associated variables (as a link between social cognitive deficits and mood disorders has been discussed [9]) and user satisfaction (as SoCoBo is designed in a very appealing and entertaining way; secondary outcomes). RehaCom^®^, on the other hand, should improve general cognition (secondary outcomes) to a higher degree than the SoCoBo intervention. Thus, the following four hypotheses can be derived:

*Hypothesis 1: In the SoCoBo group, significant improvements for measures of social cognition (primary outcomes: emotion recognition, empathy/theory of mind, social problem solving) are expected compared to RehaCom^®^*.

*Hypothesis 2: For the RehaCom^®^ group, significant improvements are expected with regard to the cognitive functioning (secondary outcomes: attention, memory, executive functions) compared to the SoCoBo group*.

*Hypothesis 3: For the SoCoBo group, the extent of improvements in socio-cognitive functioning should be positively correlated with increased life satisfaction and decreased depressive and anxiety symptoms (secondary outcomes)*.

*Hypothesis 4: Overall user satisfaction (secondary outcome) of the newly developed SoCoBo intervention should be at least comparable to RehaCom^®^*.

## Methods

### Participants

**Fig 1:**
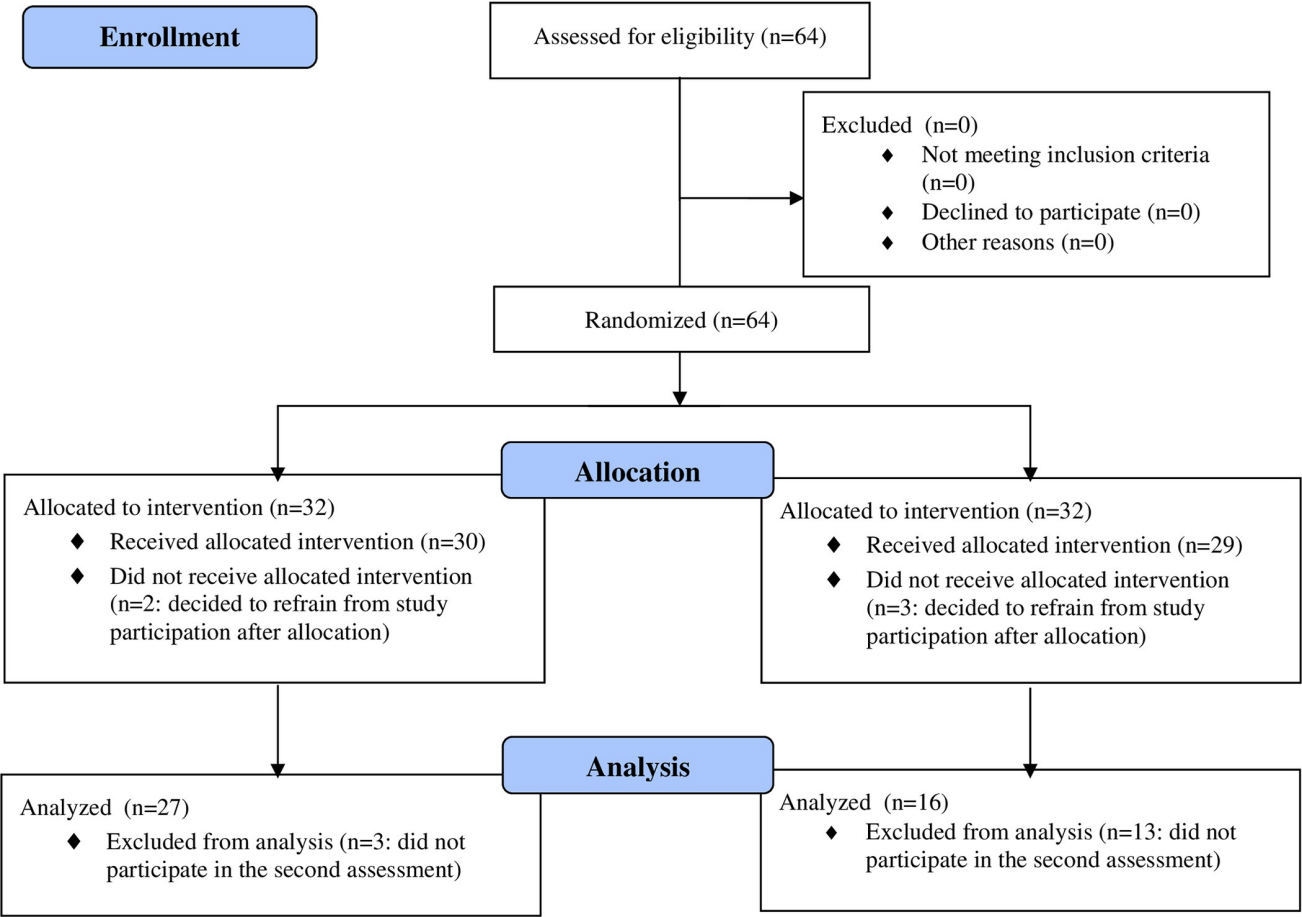
Consort 2010 flow diagram.

In total, 64 patients TBI patients recruited in Germany, Austria and Switzerland via mailing lists, newsletters, newspapers, self-help groups and from on-going on-site therapies in the Neuropsychological Therapy Centre in Bochum were randomly assigned to one of the two parallel conditions (SoCoBo: 32 participants vs. RehaCom^*®*^: 32 participants; allocation ratio: 1:1; see also participant flow diagram: Fig 1). A computerized random number generator was used for simple randomization. 59 patients participated in the pre-treatment assessment (for more details on the assessments implemented in this study see section Assessment). The sample was recruited between September 6^th^, 2021 and March 31^th^, 2023. Eventually, 43 patients completed one of the two conditions (SoCoBo: 27 participants, RehaCom^*®*^: 16 participants) including the comprehensive pre- and post-treatment assessments. All patients were offered the possibility to complete the respective other intervention after completing the condition they had been randomly allocated to. If they chose to do so, in any case, before switching to the other condition, participants underwent post assessment. Fifteen patients completed the other condition as well after finishing the first one (nine patients in the RehaCom^*®*^ group and six patients in the SoCoBo group) and were then retested (second post assessment). Eleven participants decided to terminate the online therapy prematurely (nine patients in the RehaCom^*®*^ group, two in the SoCoBo group), which corresponds to a dropout rate of 18.64 percent. Only those participants are being referred to as dropouts that at least fully completed the pre-treatment assessment. Since only two participants in the SoCoBo group terminated the therapy prematurely, it can be assumed that the participants in the SoCoBo group in particular showed a high level of compliance with the SoCoBo online therapy.

With regard to the eligibility criteria, it was ensured that the patients were actually diagnosed with a TBI (only post-acute outpatient participants were included). The age of the included patients ranged between 16 and 85 years. The participants had to have good knowledge of the German language, an estimated IQ above 80, and basic skills in using PCs (including the availability of an internet connection and an e-mail account). Progressive diseases of the central nervous system, the presence of another ABI as well as mental disorders from the psychosis / addiction spectrum, moderate to severe aphasia, an amnesic syndrome, severe manifestations of visual field impairment or neglect, and severe motor impairments in the hand/arm led to exclusion, while other comorbid mental diseases and medicated posttraumatic epilepsy were tolerated due to their frequent co-occurrence in TBI. Participation was also not possible if the patients had participated (or were still participating) in specific social skills therapy programs. If patients regularly used a program to improve cognitive deficits, they were asked to refrain from doing so during study participation. Other than that, the participants continued to receive any treatment they had started prior to enrolling for the study (e.g., neuropsychological, psychotherapeutic, pharmacological treatment). All patients who were included in this study met these previously described eligibility criteria and gave written informed consent. For minors (between 16 and 18 years of age), we also obtained written consent from parents or guardians.

Although it was pre-specified that the participants should engage in the SoCoBo online therapy for a total duration of 12 weeks, four times a week, very few of them were able to adhere to this time frame and for several participants larger gaps between the consecutive sessions occurred (e.g., due to vacation or illness), resulting in an average time of 20.04 weeks (*SD* = 9.55) for the SoCoBo group and 19.13 weeks (*SD* = 18.37) for the RehaCom^*®*^ group (*t* = .212, *p* = .833). The vast majority of participants completed the online therapy in the condition to which they were originally allocated. Two exceptions were patients who had been originally assigned to the RehaCom^*®*^ group, but for whom it was not possible to install RehaCom^*®*^ on their computer, so they were switched to the SoCoBo condition.

### Treatment

**Fig 2:**
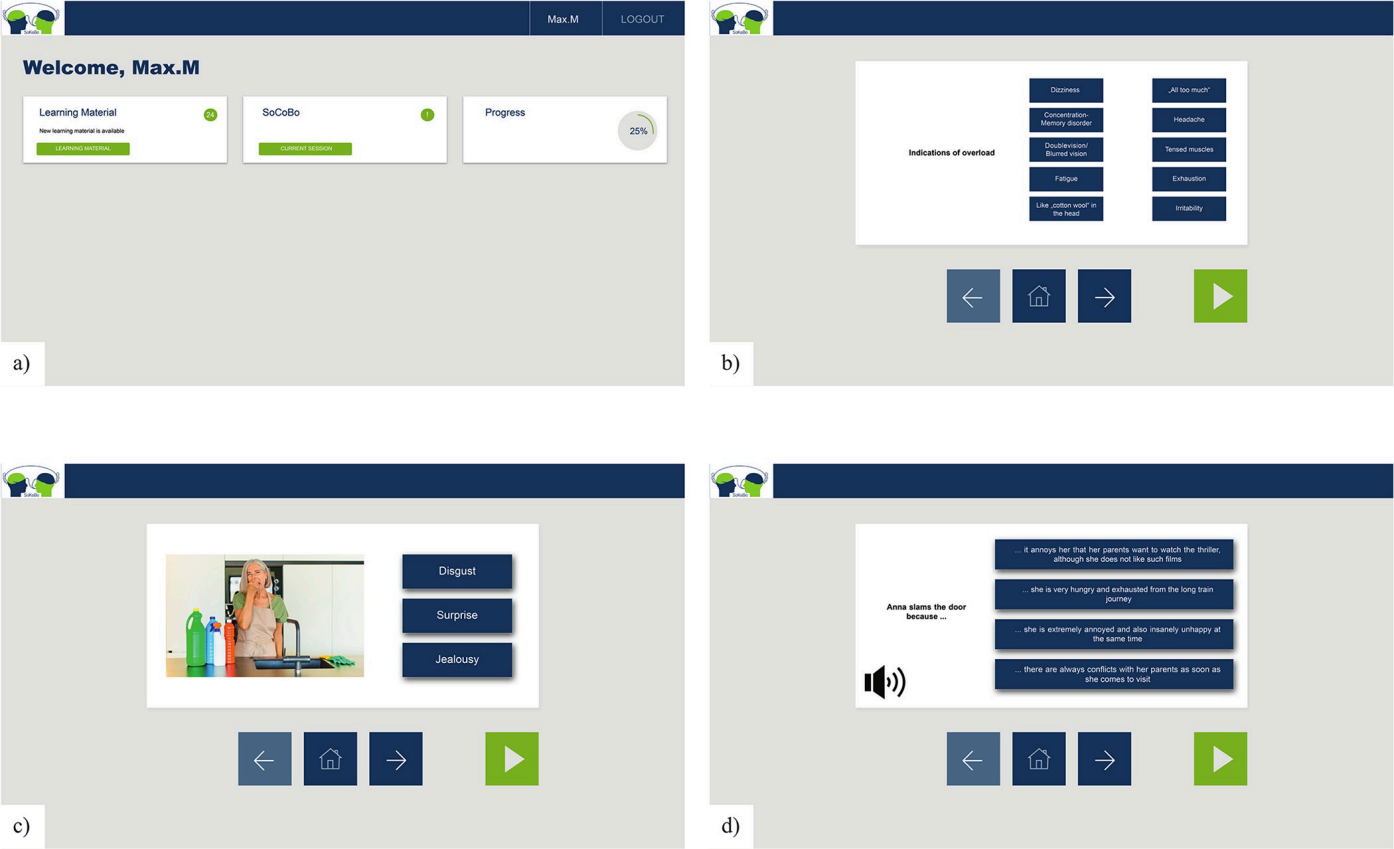
Example slides of the SoCoBo online therapy. Note. a) main menu; b) psychoeducation slide for social problem solving; c) training session including still picture of emotion expression used in the emotion recognition module; d) training session including an audio play used in the perspective taking / social problem-solving module.

**Fig 3:**
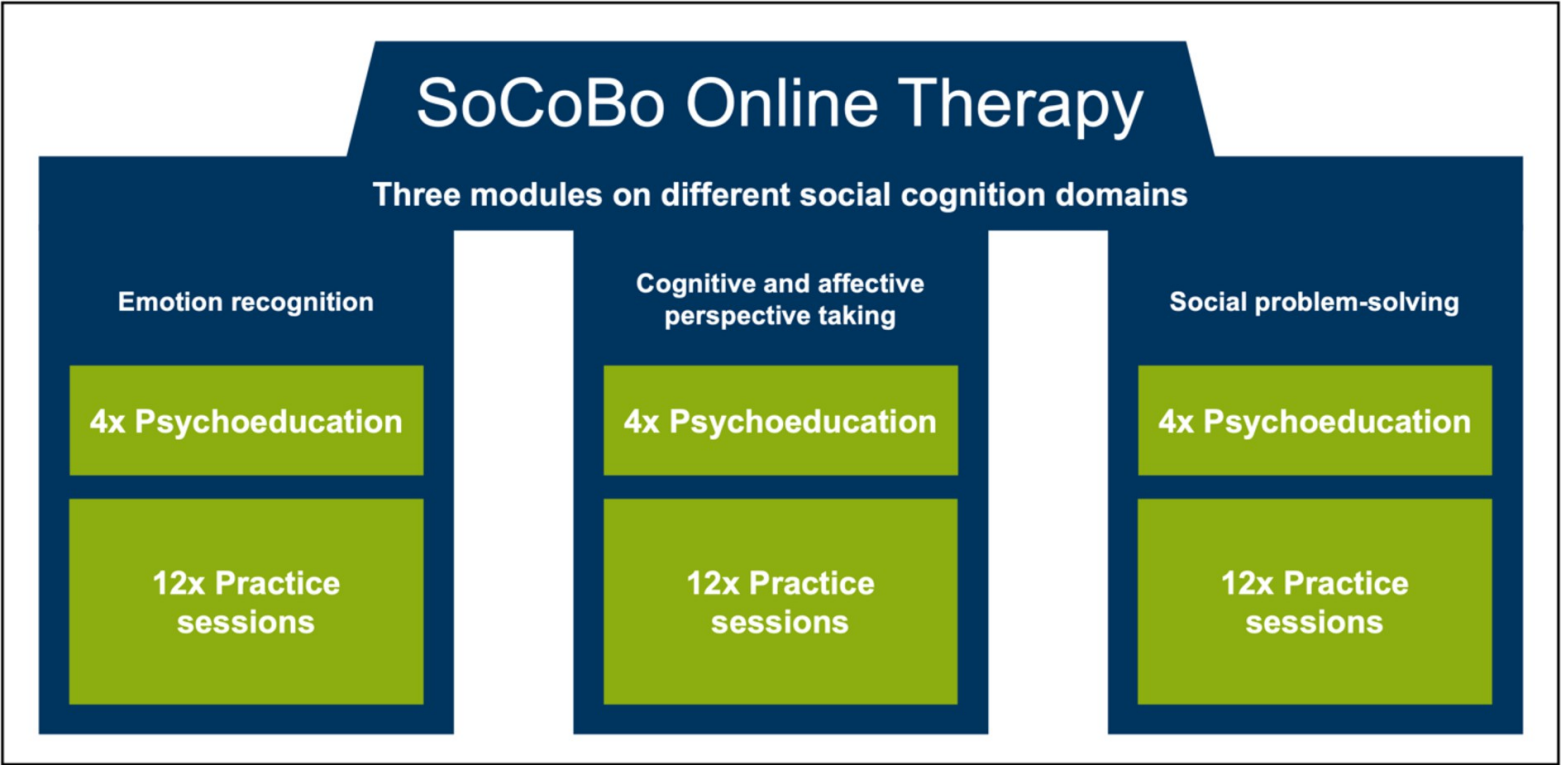
Modular structure of the SoCoBo online therapy (emotion recognition, cognitive and affective perspective taking, social problem-solving) including a distinction between psychoeducation sessions and practice sessions.

The SoCoBo online therapy (for screenshots see Fig 2)–which was programmed in cooperation with the company AppMatrix [42]–is divided into an emotion recognition module, a cognitive and affective perspective taking (ToM / empathy) module, and a social problem-solving module (16 sessions each, 48 sessions in total; see Fig 3). The total length of the SoCoBo online therapy amounts to approximately 12 weeks if four sessions are completed each week. Special features of SoCoBo involve the close interlinkage between psychoeducation and practice sessions and the provision of informative additional learning materials (presented for the participants as downloadable PDF work- and overview sheets in a submenu of SoCoBo).

In all three modules, a distinction was made between psychoeducation and practice sessions. In the psychoeducation sessions, information on the respective topic is conveyed using text, short videos and audios explaining and illustrating common problems related to emotion recognition, perspective taking and social problem-solving, and introducing strategies to deal with these impairments are described. Participants are guided through the intervention via audio clips and short videos. Important information was repeated frequently, and summaries were implemented to address deficits in memory and attention.

The practice sessions present the opportunity to transfer the knowledge acquired during the psychoeducation sessions to new stimulus material. *Emotion recognition practice sessions* involve the presentation of static and dynamic stimuli that show faces, bodies or scenes of interactions displaying a specific emotion which the participants have to identify in a multiple-choice format. When the participants select an answer, they are immediately shown the correct solution to promote an instant learning effect. The stimuli presented in the emotion recognition module were selected from various existing stimulus sets (Amsterdam Dynamic Facial Expressions Set–ADFES [43]; Atkinson stimulus set [44, 45]; The Bochum Emotional Stimulus Set–BESST [46]; EU-Emotion Stimulus Set [47, 48]; FACES stimulus set [49]; Karolinska Directed Emotional Faces–KDEF [50]) and few stimuli were additionally created by the research team (for more information on the emotion recognition stimuli used see S1 Appendix).

With regard to the *cognitive and affective perspective taking (ToM / empathy)* and *social problem*-*solving* practice sessions, audio plays and written scenarios were created depicting social interactions that can be assigned to the superordinate categories of "surprise", "joyful occasion", "compliment", "misunderstanding", "conflicts”, and "faux pas" (for more information on the audio plays and written scenarios used see S2 and S3 Appendices). Following a standardized structure, the participants have to answer multiple-choice questions after the audio plays / written scenarios that relate to how the protagonists in the audio plays / written scenarios feel, what they might think and how they should deal with the presented situation. In general, after choosing a response, for all questions involving a correct answer immediate feedback is provided in the cognitive and affective perspective taking (ToM / empathy) as well as in the social problem-solving practice sessions.

RehaCom^®^ (by Hasomed), which is the program used as control intervention in the context of this RCT study, provides cognitive remediation therapy addressing problems in attention, memory and executive functions. Several studies have already demonstrated the efficacy of RehaCom^®^ for the treatment of cognitive impairments in TBI patients [51, 52]. For the RCT study, based on different RehaCom^®^ exercises (attention: sustained attention and divided attention / memory: verbal memory and topological memory/executive function: arithmetic practice and logical reasoning), three different modules were assembled, parallelizing the modular structure of the SoCoBo intervention. To allow for maximum comparability, the participants in the RehaCom^®^ condition also completed psychoeducation sessions on memory, attention and executive functions that were created by the research team with the help of a homepage construction kit used for creating the SoCoBo sessions, as there would otherwise have been no psychoeducation in RehaCom^®^.

### Assessment

The comprehensive online pre-post assessment (2–3 hours each) included a series of questionnaires and (neuro-)psychological tests. When possible, different versions (A/B) of the respective tests were used for pre- and post-assessment. The assessment instruments used can be found in Table 1. Descriptions of the tools used for assessing social cognition, general cognition and clinical data are presented in more detail in S4 Appendix. In addition to the instruments presented in Table 1, a self-designed questionnaire was administered post-treatment to assess individual feedback and satisfaction with regard to the study participation and the SoCoBo online therapy / RehaCom^®^ program (see S5 Appendix).

**Table 1:** Instruments used in pre- and post-assessment of the randomized controlled trial.

Assessed domain	Names of the instruments and subscales	Authors
Social Cognition (primary outcomes)		
	Emotion Recognition Index (ERI)	Scherer & Scherer [53]
Facial emotion recognition	ERI (faces)	
Vocal emotion recognition	ERI (voices)	
Videobased emotion recognition	Geneva Emotion Recognition Test-Short form (GERT-S)	Schlegel & Scherer [54]
	German version of the Interpersonal Reactivity Index (IRI)	Paulus [55]
Affective Empathy	Empathic Concern	
Affective Empathy	Personal Distress	
Cognitive Empathy	Fantasy	
Cognitive Empathy	Perspective taking	
Social Competencies	The Inventory for Social Competencies—Short form (ISK-K)*	Kanning [56]
	Social orientation	
	Offensiveness	
	Self-monitoring	
	Reflexibility	
	Social Cognition Test Battery	Channon & Crawford [57]
Theory of Mind Mentalistic Interpretation	Part M	
Social Problem-Solving Social Problem Resolution	Part B	
Social Problem-Solving Social Problem Fluency	Part F	
Alexithymia	The Toronto Alexithymia Scale (TAS-20)	Bagby et al. [58]
General Cognition (secondary outcomes)		
Short- and midterm memory	Auditory Verbal Learning Test (AVLT)	Helmstaedter et al. [59]
Executive functions	The Regensburg word fluency test (RWT)	Aschenbrenner et al. [60]
Executive functions	Wechsler Memory Scale: Digit span	Wechsler [61]
Executive functions	Scale for the Assessment of Action, Planning and Problem-solving Impairments (HPP-S)	Gauggel & Deckersbach [62]
Executive functions	German version of the Stroop Test (FWIT)	Bäumler [63]
Processing speed	German version of the Stroop Test (FWIT)	Bäumler [63]
Vocabulary intelligence	German vocabulary intelligence test (MWT-B)**	Lehrl [64]
Mental Health (secondary outcomes)		
Depression	Rasch-based Depression Screening (DESC)	Forkmann et al. [65]
Social interaction anxiety	Social Interaction Anxiety Scale (SIAS)	Heinrichs et al. [66]
State anxiety	State-Trait Anxiety Inventory (STAI)	Spielberger [67]
Trait anxiety	State-Trait Anxiety Inventory (STAI)	Spielberger [67]
Life Satisfaction	Questionnaire on Life Satisfaction (FLZ)	Fahrenberg et al. [68]

Note.

*For this instrument also a proxy assessment was sent to relatives of the TBI patients

**This instrument was used only in the first assessment

### Study design

An RCT was implemented (following the CONSORT 2010 guidelines / checklist for reporting parallel group randomized trials [69], see S1 Checklist) with TBI patients being randomly allocated to an experimental (SoCoBo) and a control condition (RehaCom^®^). The RCT was registered as a clinical trial in the German Clinical Trials Register (DRKS00032243). Correspondingly, all authors confirm that all ongoing and related trials for this intervention are registered. The delay of the registration was due to the immense preparatory work (programming of the online therapy, patient recruitment) that was necessary for the start of the study in 2020. The study design did not allow for blinding with regard to the two conditions for the participants nor for the psychologists who supervised the study. The random allocation sequence, generated by author SR, was not concealed, but it was strictly followed without any deviation. The participants were enrolled by authors SR and TL, and the same authors assigned the participants to the interventions.

The participants in both conditions were asked to complete four sessions per week for 12 weeks. The duration of a single session in SoCoBo and RehaCom^®^ is approximately 30 minutes. The order of the presented sessions/modules in the SoCoBo and RehaCom^®^ condition was the same for everyone who participated in the study (1. emotion recognition, 2. cognitive and affective perspective taking, 3. social problem-solving). This ensured optimal parallelization of the procedure for each participant and was also theoretically motivated as it was assumed that emotion recognition and perspective taking provide basic building blocks for the ability to solve social problems [18]. For both conditions, regular telephone conversations were conducted with the patients every two weeks, allowing a reflection on the content of the various sessions of the respective conditions with the help of trained research assistants. In general, care was taken to ensure that the timing and conditions of assessments and treatment (including the order of the presented tests / sessions) were the same between both groups.

The overall study design was approved by the Ethics Committee of the Faculty of Psychology of the Ruhr University Bochum (EFP-RUB; approval number: 436). The research was completed in accordance with the Helsinki Declaration. Any human data included in this manuscript was obtained in compliance with regulations of the Ruhr University Bochum. It could be realized as initially planned during the implementation phase. A deviation from the original study protocol (see S1 File) results from the fact that, contrary to the initial plan, no stroke patients were recruited for this RCT, but instead the focus was on traumatic brain injury patients in order to keep the overall study procedure within a feasible framework. Another adjustment in comparison to the original study protocol relates to the inclusion criteria, with participants aged 16 or 17 were also included in order to acquire a larger sample, and deficits in social cognition not necessarily had to be reported in order to be included. In addition, some assessments were not implemented as originally planned due to the need to switch to online assessments in connection with the COVID-19-pandemic. Also due to the contact restrictions in the context of the COVID-19-pandemic, audio plays instead of videos had to be created for the exercise sessions. Furthermore, the total number of weeks was reduced from 16 to 12 weeks and the interlocking of the modules (and the choice of the RehaCom^®^ modules) was slightly changed compared to the original plan in order to enhance feasibility of the study procedure. No harms or unintended effects appeared in both groups. The trial ended in March 2023 because of the end of the planned duration of the trial due to the ending of funding.

### Data analysis

Data analysis, conducted with SPSS 29, comprised ANOVAs, MANOVAs and partial correlations with age, intelligence and gender being used as covariates in all of these analyses. A G*Power (version 3.1.9.2) compromise power analysis including 43 participants revealed a power of .92 for conducting a repeated measures ANOVA and .72 for conducting a repeated measures MANOVA (with effect sizes of .25 being assumed). In rare cases, missing data existed with regard to individual questionnaires and tests as they could not be completed or analyzed due to technical difficulties or misunderstood instructions. Overall, therefore data for 34–42 participants exist, depending on the questionnaire and test. For the proxy assessment questionnaire (ISK), significantly less data was collected overall (31 relatives filled out the questionnaire before the start of the intervention, 19 after the end of the intervention). A significance level of *p* < 0.05 was chosen in this study.

For Hypothesis 1 and 2, repeated measures ANOVAs and MANOVAs with group (SoCoBo vs. RehaCom^®^) as between-subjects factors and time of assessment (pre-post intervention) as within-subject factor were calculated, including age, gender and intelligence as covariates. Exploratively, with regard to Hypothesis 1, a repeated measures ANOVA was also used to test whether participants who first completed the RehaCom^®^ condition and then switched to the SoCoBo condition also improved in social cognition after completing the SoCoBo condition. In order to determine between which measurement time points improvements occurred, planned contrasts were computed. More specifically, in SPSS the planned contrast "deviation" was selected, which compares the mean of each factor level (except the reference category, in this case the first measurement point) with the mean of all factor levels (overall mean). For Hypothesis 3, a difference score was calculated for each patient between the post- and pre-treatment values in order to gauge the increase between pre- and post-assessments. Partial correlations were then calculated between the pre-post difference and the questionnaires measuring life satisfaction, depression and anxiety assessed post-treatment (age, gender and intelligence were again included as covariates). With regard to Hypothesis 4, the item “I am satisfied with the program” from the self-generated feedback questionnaire was used to analyze the user satisfaction in the two groups (SoCoBo vs. RehaCom^®^) and a univariate analysis of variance with age, gender and intelligence as covariates was calculated.

## Results

An overview of the demographic data of the participants who completed the respective conditions can be found in Table 2. Information on the clinical data (life satisfaction, depression, and anxiety) during the pre- and post-treatment assessment is presented in Table 3. Forty-two out of 59 patients took medication (20 in the SoCoBo group, 22 in the RehaCom^*®*^ group, which indicates that in both groups the use of medication was comparable), particularly antidepressant (15 patients) and antiepileptic (again 15 patients) agents. Furthermore, 39 out of 59 participants received neuropsychological therapy and 21 out of 59 participants received psychotherapy. For the patients who completed the pre- and post-treatment assessment, on average, the TBI occurred 83.14 months ago (*SD =* 72.64, min = 18, max = 293, *N =* 43; SoCoBo group: 90.30 months ago, *SD* = 76.96, RehaCom^*®*^ group: 71.06 months ago, *SD* = 65.26; *t* = .836, *p* = .408). The average time these participants remained in a coma following the event–which can be regarded a proxy measure for illness severity–was 30.58 days (*SD =* 48.03, *N* = 31; SoCoBo group: 29.05 days, *SD* = 45.07, RehaCom^*®*^ group: 33.36 days, *SD* = 55.20; *t* = -.235, *p* = .815). This indicates that illness severity appears to have been comparable in both groups. With regard to the side the injury occurred, it can be stated that 13 participants had right-sided injury, 12 participants had left-sided injury, 8 participants had central injury and 8 participants had bilateral injury, with this information only being available for 41 participants.

**Table 2:** Demographic data (frequencies, means and standard deviations) for the participants in the SoCoBo group and the RehaCom® group who have completed the pre- and post-treatment assessment.

	SoCoBo group (*N* = 27)	RehaCom® group (*N* = 16)	*F/chi*^*2*^ values	*df*	*p*-values*
Age in years, *M (SD)*	44.11 (16.78)	46.38 (16.47)	.185	1, 41	.669
Gender (f / m)	10 / 17	3 / 13	1.593	1	.207
IQ (MWT), *M (SD)*	106.15 (15.26)	103.06 (10.08)	.516	1, 40	.477

*Note*. f = female; m = male; *M* = Mean; MWT = German vocabulary intelligence test; *SD* = Standard deviation

*the *p-*values indicate whether the two groups differ significantly from each other with regard to the respective variables

**Table 3:** Mean raw scores and standard deviations (SoCoBo group and RehaCom® group) for the clinical measures at the pre- and post-treatment assessment.

Outcomes Measures	Pre-treatment				
	SoCoBo group	RehaCom® group	*F-*values	*df*	*p-*values
	(*N* = 26–27)	(*N* = 29)			
	*M*(*SD*)	*M*(*SD*)			
Depression: DESC	13.04 (6.72)	12.00 (9.44)	.340	1,51	.562
Life Satisfaction: FLZ	226.48 (36.27)	229.38 (44.55)	.003	1,51	.954
Social Interaction Anxiety: SIAS	32.59 (14.58)	25.07 (11.81)	4.25	1,51	.044*
State Anxiety: STAI X1	37.59 (10.05)	38.83 (12.05)	.463	1,51	.499
Trait Anxiety: STAI X2	48.85 (11.75)	46.21 (11.35)	.456	1,50	.502
Outcomes Measures			Post-treatment		
	SoCoBo group	RehaCom® group	*F-*values	*df*	*p-*values
	(*N* = 26)	(*N* = 15)			
	*M*(*SD*)	*M*(*SD*)			
Depression: DESC	10.96 (5.92)	11.00 (8.73)	.000	1,39	.987
Life Satisfaction: FLZ	227.69 (37.49)	245.53 (44.32)	1.89	1,39	.178
Social Interaction Anxiety: SIAS	28.08 (10.26)	25.47 (13.22)	.497	1,39	.485
State Anxiety: STAI X1	33.54 (11.59)	30.73 (9.21)	.642	1,39	.428
Trait Anxiety: STAI X2	45.50 (10.46)	41.73 (11.89)	1.12	1,39	.297

*Note*. DESC = Rasch-based Depression Screening; FLZ = questionnaire on general life satisfaction; *M* = Mean; *SD* = Standard deviation; SIAS = Social Interaction Anxiety Scale; STAI = State-Trait Anxiety Inventory

*significant *p* < .05

The means and standard deviations of the various measures and respective dependent variables of social cognition and general cognition for the SoCoBo group and the control group relevant to Hypothesis 1 and Hypothesis 2 are presented in Table 4.

**Table 4:** Mean raw scores or percent correct* and standard deviations (SoCoBo group and RehaCom® group) for the measures of social cognition and general cognition used in pre- and post-treatment assessments.

Outcomes Measures	SoCoBo group (*N* = 19–26)		RehaCom® group (*N* = 14–17)	
	Pre-treatment *M(SD*)	Post-treatment *M(SD*)	Pre-treatment *M(SD*)	Post-treatment *M(SD*)
Social Cognition				
Emotion recognition: ERI (faces); % correct	62.70 (16.62)	72.13 (11.69)	62.40 (9.65)	63.87 (8.03)
Emotion recognition: ERI (voices); % correct	55.35 (17.60)	56.96 (17.13)	53.80 (16.00)	56.40 (12.57)
Emotion recognition: GERT; % correct	18.45 (6.95)	20.91 (6.38)	18.07 (5.78)	18.00 (6.85)
Empathy: IRI				
Empathic Concern	13.71 (2.54)	14.12 (2.87)	13.21 (3.18)	12.07 (2.94)
Personal Distress	13.71 (3.56)	12.42 (3.40)	11.72 (3.41)	11.67 (3.42)
Fantasy	11.21 (3.39)	12.35 (2.91)	10.93 (3.68)	11.20 (3.28)
Perspective taking	12.25 (3.37)	13.42 (2.66)	11.83 (3.82)	12.27 (3.71)
Total**	36.67 (7.17)	39.63 (6.53)	37.07 (7.56)	35.53 (8.11)
Social Competencies: ISK-K				
Social orientation	24.53 (4.31)	25.00 (3.80)	25.47 (5.60)	26.27 (4.99)
Offensiveness	18.89 (4.32)	19.37 (4.03)	21.13 (3.29)	20.67 (2.85)
Self-monitoring	16.89 (4.04)	17.42 (3.81)	20.00 (4.05)	20.47 (4.44)
Reflexibility	19.84 (2.61)	19.21 (2.64)	18.40 (2.61)	18.53 (2.30)
ToM: Social Cognition Test Battery Part M				
Quality of interpretation score	1.58 (.33)	1.63 (.47)	1.61 (.49)	1.56 (.54)
Selection of alternatives score	1.91 (.29)	1.89 (.40)	1.80 (.37)	1.78 (.42)
Total	23.92 (3.76)	24.42 (4.53)	23.12 (5.43)	23.44 (5.16)
Social Problem-Solving: Social Cognition Test Battery Part B				
Solution quality score	1.39 (.36)	1.56 (.32)	1.36 (.40)	1.40 (.47)
Total	6.88 (1.82)	7.73 (1.76)	6.81 (2.01)	7.06 (2.27)
Social Problem-Solving: Social Cognition Test Battery Part F				
Detection of awkward ness	.73 (.29)	.69 (.25)	.71 (.24)	.71 (.25)
Solutions fluency total score	2.33 (.81)	2.37 (.78)	2.38 (.74)	2.53 (.71)
Socially sensitive and practically effective solutions score	1.09 (.54)	1.11 (.46)	1.18 (.73)	1.20 (.35)
Total	23.00 (5.39)	23.40 (5.12)	22.73 (3.79)	22.67 (4.69)
Alexithymia: TAS-20 Total	55.71 (12.63)	50.29 (11.30)	53.82 (14.35)	45.00 (20.21)
General Cognition				
Regensburg word fluency test				
Formal lexical	8.92 (3.85)	8.54 (3.99)	10.00 (4.46)	10.53 (4.34)
Semantic	17.50 (7.05)	17.54 (5.90)	18.60 (6.17)	18.40 (5.90)
Semantic Change	12.04 (3.52)	11.96 (3.55)	11.27 (3.65)	11.20 (3.67)
Digit Span Forwards; seconds	8.00 (2.12)	8.00 (1.74)	7.88 (1.93)	7.56 (1.86)
Digit Span Backwards; seconds	6.23 (2.34)	6.62 (2.39)	7.00 (2.28)	7.13 (2.45)
Auditory Verbal Learning Test***				
AVLT 1–5	49.46 (11.43)	48.92 (11.93)	50.19 (13.43)	47.13 (13.83)
AVLT 5–6	2.31 (1.96)	2.23 (2.18)	1.75 (1.53)	1.56 (2.13)
AVLT 5–7	2.88 (2.46)	2.77 (2.22)	1.81 (1.56)	1.19 (1.97)
Stroop Test; seconds				
Color word reading	48.11 (17.47)	46.15 (17.34)	39.19 (9.32)	40.63 (11.53)
Color naming	62.98 (18.95)	61.82 (24.59)	54.37 (12.04)	54.57 (10.99)
Interference	100.73 (41.40)	94.82 (35.79)	85.78 (18.89)	80.15 (16.29)
HPP-S Total	11.75 (4.48)	10.92 (4.93)	9.36 (4.07)	9.29 (4.48)

*Note*. ERI = Emotion Recognition Index; GERT = Geneva Emotion Recognition Test-Short form; HPP-S = the Scale for the assessment of action, planning and problem-solving impairments; IRI = Interpersonal Reactivity Index; ISK-K = Inventory for Social Competencies–Short form; *M* = Mean; *SD* = Standard deviation

* If not explicitly stated otherwise in the respective lines, the scores presented reflect raw scores

** perspective taking subscale + empathic concern subscale + fantasy subscale

***AVLT 1–5 reflects learning performance, AVLT 5–6 reflects interference effects, AVLT 5–7 reflects retrieval performance after delay

With regard to two social cognition measures (primary outcomes), facial emotion recognition assessed with the Emotion Recognition Index (ERI, % correct) and self-rated empathy assessed with the German version of the Interpersonal Reactivity Index (IRI, total empathy score calculated as summed score for the three subscales perspective taking, empathic concern and fantasy), significant interaction effects between the group and the time of assessment were revealed (ERI faces: *F*(1, 33) = 4.245, *p* = .047, *η*^*2*^_*p*_ = .114; IRI: *F*(1, 34) = 4.359, *p* = .044, *η*^*2*^_*p*_ = .114).

**Fig 4:**
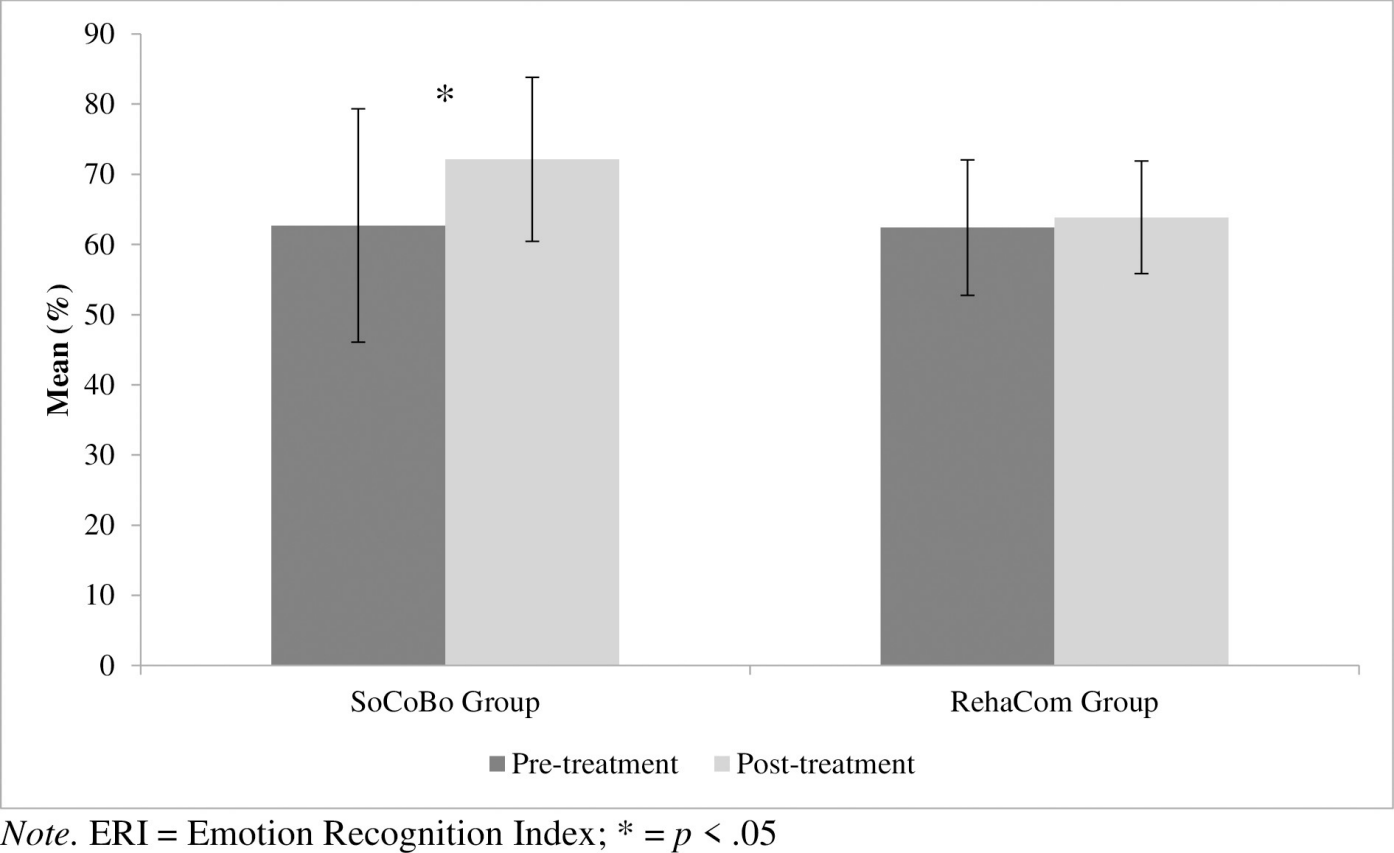
Mean percent correct and standard deviations (SoCoBo group and RehaCom^®^ group) for emotion recognition (ERI faces) at pre- and post-treatment assessment. *Note*. ERI = Emotion Recognition Index; * = *p* < .05.

**Fig 5:**
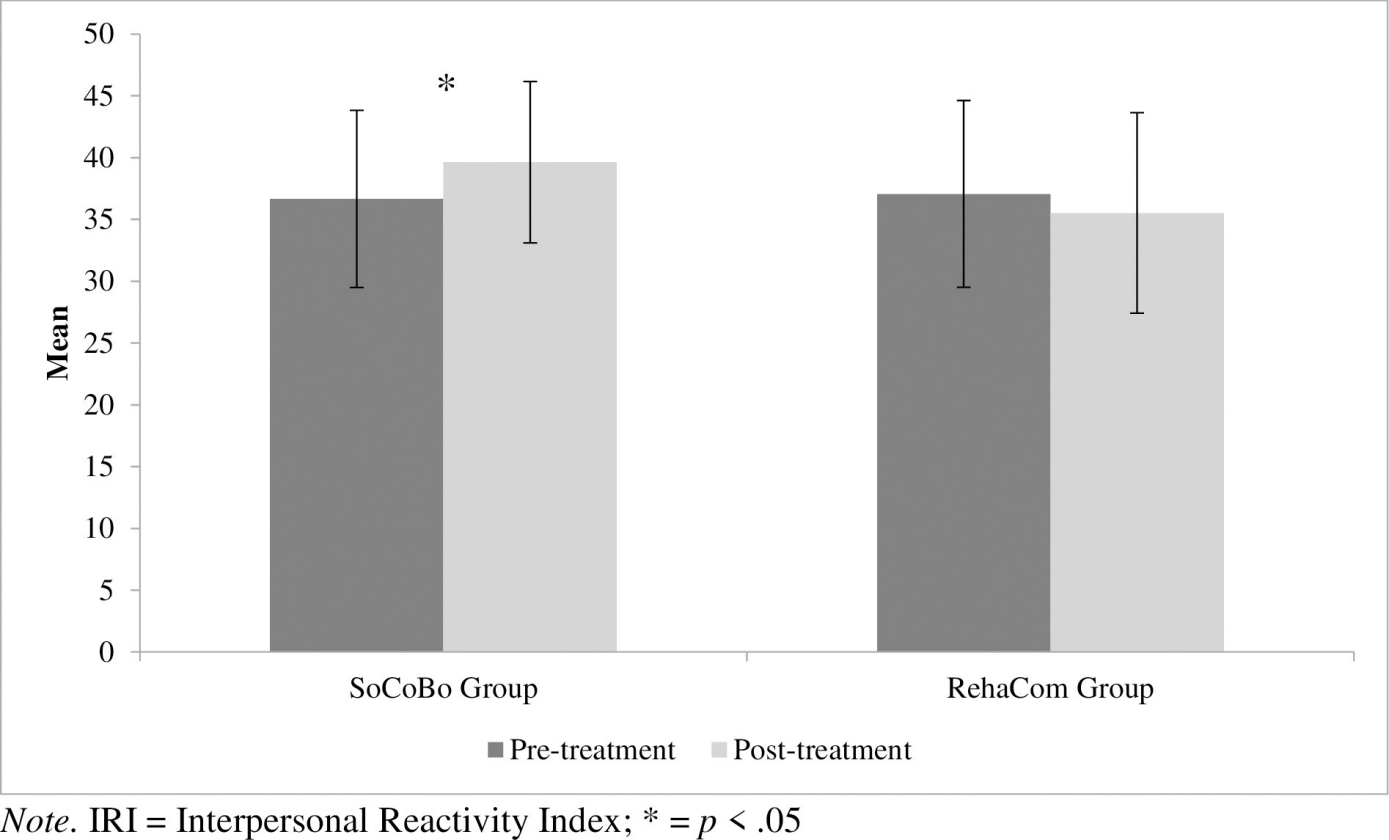
Mean raw scores and standard deviations (SoCoBo group and RehaCom^®^ group) for the empathy total score (IRI) including the subscales perspective taking, empathic concern and fantasy at pre- and post-treatment assessment. *Note*. IRI = Interpersonal Reactivity Index; * = *p* < .05.

To resolve the significant group * time of assessment interactions, post-hoc analyses of variances were computed separately in the two intervention groups, revealing a significant increase of correct response for ERI faces from the pre- to the post-treatment assessment in the SoCoBo group (*F*(1, 23) = 11.627, *p* = .002, *η*^*2*^_*p*_ = .336), but not in the RehaCom® group (*F*(1, 14) = 0.397, *p* = .539, *η*^*2*^_*p*_ = .028). In the case of IRI there is again a specific increase of self-rated empathy in the SoCoBo group (*F*(1, 24) = 4.852, *p* = .037, *η*^*2*^_*p*_ = .168), but no significant change from pre- to post-interventional assessment in the RehaCom® group (*F*(1, 14) = 0.932, *p* = .351, *η*^*2*^_*p*_ = .062). Importantly, the results can be replicated for both significant social cognition outcomes if an ANCOVA is calculated as an alternative method of analysis, comparing the group’s performance in the post-tests and including the pre-test (and age, gender and intelligence) as a covariate (ERI faces: *F*(1, 32) = 5.320, *p* = .028, *η*^*2*^_*p*_ = .143; IRI: *F*(1, 33) = 4.891, *p* = .034, *η*^*2*^_*p*_ = .129). The corresponding means are shown in Table 4 (for a graphical representation of the mean values and standard deviations see Figs 4 and 5).

The effect for the ERI faces (but not the IRI) is also replicated for the subgroup which started with the RehaCom^®^ condition and then switched to the SoCoBo condition after completing the RehaCom^®^ condition (*N* = 8, one case excluded because of missing values). The means and standard deviations for this subgroup were 61.38 (*SD* = 9.64) pre-treatment, then 62.25 (*SD* = 7.69) after completing the RehaCom^®^ condition and finally 71.88 (*SD* = 15.10) after the SoCoBo condition (*F*(1, 7) = 7.097, *p* = .032, *η*^*2*^_*p*_ = .503). As planned contrasts show, the mean of the second measurement is not different from the first measurement (*F*(1, 7) = .140, *p* = .720, *η*^*2*^_*p*_ = .020), while the third measurement yielded significant increases (*F*(1, 7) = 6.012, *p* = .044, *η*^*2*^_*p*_ = .462). This means that the main increase occurred after the SoCoBo, but not after the RehaCom^®^ intervention.

With regard to the other measures of social cognition (ERI voices subscale; Geneva Emotion Recognition Test-Short form [GERT-S]; Inventory for Social Competencies–Short form [ISK-K] involving the subscales Social orientation, Offensiveness, Self-monitoring, Reflexibility; the Social Cognition Test Battery involving different analyses for the subtests Mentalistic Interpretation Task [Part M], Social Problem Resolution Task [Part B], Problem Fluency Task [Part F]; Toronto Alexithymia Scale [TAS-20]) no significant interactions between the groups and the time of assessment were revealed (all *p*-values > .05; see S6 Appendix for the presentation of the non-significant main effects and time*group interactions). In general, it should be noted with regard to Hypothesis 1 that an analysis of the proxy assessments was not performed due to the overall small number of respondents.

With regard to Hypothesis 2 relating to general cognition (secondary outcomes), it can be stated that no significant interaction effect between the groups and time of measurement was revealed (all *p*-values > .05). This means that the participants of the RehaCom^*®*^ group did not improve their cognitive performance in comparison to the SoCoBo group (Regensburg word fluency test [RWT], including the subtests Formal lexical, Semantic, Semantic Change; Digit Span Forwards / Backwards; Auditory Verbal Learning Test [AVLT] including AVLT 1–5 reflecting learning performance, AVLT 5–6 reflecting interference effects and AVLT 5–7 reflecting retrieval performance after delay; Stroop Test including the subtests Color word reading, Color naming and Interference; Scale for the Assessment of Action, Planning and Problem-solving Impairments [HPP-S]). Moreover, there was no main effect of time meaning that both groups did not change over time (again all *p*-values > .05). The respective non-significant results of the statistical analyses calculated in association with Hypothesis 2 are presented in S6 Appendix.

With regard to Hypothesis 3 relating to possible mental health effects (secondary outcomes), the variables ERI faces and IRI were included due to the significant improvement of these variables in the SoCoBo group compared to the RehaCom^®^ group. The only significant correlation was an increase in empathy being associated with increased life satisfaction post-treatment (*r* = .46, *p* = .034; see Table 5).

**Table 5:** Partial correlations (covariates: Age, intelligence, gender) between the pre-post ERI faces difference / pre-post IRI difference and the questionnaires on life satisfaction, depression and anxiety at post-treatment assessment.

	FLZ	DESC	SIAS	STAI X1	STAI X2
			
ERI faces	-.09	.17	-.05	-.19	.01
IRI	.46*	.11	.04	-.22	-.31

*Note*. DESC = Rasch-based Depression Screening; ERI = Emotion Recognition Index; FLZ = questionnaire on general life satisfaction IRI = Interpersonal Reactivity Index; STAI = State-Trait Anxiety Inventory; SIAS = Social Interaction Anxiety Scale; * *p* < .05

For Hypothesis 4 relating to user satisfaction (secondary outcome) no difference in satisfaction between the SoCoBo and RehaCom^®^ programs was observed (*F*(1,29) = 3.070, *p* = .090, *η*^*2*^_*p*_ = .096), with the mean values being descriptively higher in the SoCoBo group (SoCoBo: *N* = 21, Mean = 3.38, *SD* = .50; RehaCom^®^: *N* = 13, Mean = 3.08, *SD* = .64). In the SoCoBo sample, one case is missing in this analysis due to missing values in the covariates. The mean raw scores and standard deviations for all self-generated feedback questionnaire items that were not relevant for Hypothesis 4 can be found in S7 Appendix.

With regard to the data analyses, it is important to note that for five patients who completed the pre- and post-treatment assessments (and who are accordingly included in the statistical analyses), the post-treatment assessment had to be carried out before all therapy sessions were completed. The reasons for this were in four cases that an earlier appointment for the post-treatment assessment was requested because of a lack of motivation to continue study participation and in one case that the deadline for inclusion in the final data analysis would otherwise have been exceeded. Accordingly, these participants only completed between 50 and 77 percent of the initially planned sessions.

## Discussion

### Summary of the main findings

After several weeks of treatment, the SoCoBo group, but not the RehaCom^®^ group showed significant improvement in important domains of social cognition (facial emotion recognition and self-rated empathy, Hypothesis 1). Hypothesis 1 can thus be partially confirmed for two central measures of social cognition. Crucially, therefore, important significant results have been revealed in the primary outcomes, i.e. the outcomes that are considered especially important with regard to the SoCoBo efficacy evaluation, since SoCoBo was designed to especially address deficits in specific subdomains of social cognition. Contradicting Hypothesis 2, it was revealed that the RehaCom^*®*^ group compared to the SoCoBo group did not improve in cognitive performance (secondary outcome). With regard to Hypothesis 3, it was shown that the increase in empathy in the SoCoBo group was related to more life satisfaction (secondary outcome) at the post-treatment assessment. Thus, this hypothesis can be considered confirmed for this particular correlation. Finally, in line with Hypothesis 4 the level of satisfaction with SoCoBo (secondary outcome) was at least as high as with RehaCom^®^.

### Interpretation of the main findings

In general, the fact that significant changes were shown regarding (facial) emotion recognition, but not regarding behavior-associated areas of social cognition (social problem solving) corresponds to the current state of research on computerized treatment of social cognition deficits as presented in the systematic review by Lohaus et al. [38], now also demonstrated for patients with TBI.

The fact that no effects were observed regarding voice-based emotion recognition is plausible given that SoCoBo itself does not present any audio material in the emotion recognition module from which the emotion is to be identified. The finding that no effects were found for video-based emotion recognition (assessed with the GERT) is probably due to the fact that in SoCoBo significantly less video material was presented compared to static pictures (overall, only 32.5 percent of the stimuli used in the emotion recognition practice sessions were video based, see S1 Appendix). Regarding the improvements found with regard to facial emotion recognition in this study, these findings might in principle also be explained by improvements in attentional functions, since attentional processes are to be regarded as an important component of emotion recognition. However, as sociocognitive improvement was not observed in the control condition which specifically targeted improvement in attentional and other higher-order cognitive functioning), it can at least be stated that improvement of attentional functions cannot be the sole factor driving the observed changes.

With regard to behavior-associated social cognitive domains, presumably, to achieve at least a certain effect in this regard, it might be crucial to add more therapeutic assistance to the framework of the SoCoBo online therapy that goes beyond phone conversations taking place every two weeks. This would be particularly plausible given that significant results with regard to ToM and social behavior emerged in the study by Westerhof-Evers et al. [39], with these modules being implemented on-site and not computerized. Importantly, a therapist’s involvement in computerized interventions also appears to be a key element to generalization [70].

It is striking that no improvements in general cognition could be demonstrated in the RehaCom® group, although these effects have already been shown in previous studies [51, 52]. A possible reason could be that the patients were less motivated because they initially applied for the social cognition intervention. Overall the effects of cognitive remediation therapies have been critically discussed [71, 72].

Moreover, with regard to the improvement of empathy in the SoCoBo group compared to the RehaCom^®^ group, this finding also emerged in another study evaluating a computerized social cognition intervention [73] including the same instrument for assessing self-rated empathy (IRI). In this study [73], a positive effect on self-rated empathy was shown implementing a specific emotion recognition intervention. Thus, it might be possible that the emotion recognition module in SoCoBo online therapy was also responsible for improving empathy in addition to (facial) emotion recognition. However, it is also likely that the perspective taking module of SoCoBo had a decisive impact, as it was designed to also improve the affective part of perspective taking, which can be equated with cognitive empathy being also assessed by the IRI, as reflected in the subscales "Fantasy" and "Perspective Taking".

Moreover, the finding that improvements in empathy are also associated with higher life satisfaction scores corresponds well to the findings of studies that have already shown that empathy is associated with life satisfaction [74]. It is also remarkable that the SoCoBo online therapy is associated with at least similarly high user satisfaction compared to RehaCom^®^. This finding is particularly important to note given that RehaCom^®^ has been used frequently in clinical practice for several years.

Although improvements in (facial) emotion recognition and self-rated empathy—which can be regarded important subdomains of social cognition—have been shown in this study, it cannot be ultimately concluded that SoCoBo is associated with improvements in social cognition in general, as social cognition consists of many more subdomains than just emotion recognition and empathy. With regard to the significant results revealed, it should moreover be noted that these are only statistically significant results, but not necessarily also clinically significant results (i.e. significance of the revealed changes for patients’ everyday lives). It is not known to what extent a certain change measured with the ERI and the IRI has clinical significance, which means that the question of the clinical significance of the findings remains open at this point and must be addressed in future studies. In particular, regarding self-reports such as the TBI it has to be kept in mind that patients’ metacognitive abilities and self-awareness might be impaired [75]. Furthermore, since several statistical tests were carried out for the respective social cognition domains, it should also be noted that a correction for multiple testing must be considered at this point to reduce the likelihood of a Type I error. If, for example, the Bonferroni correction is applied, the significant findings described earlier in relation to social cognition disappear. However, one should also keep in mind that the Bonferroni correction can be considered a rather conservative correction method, which in turn will increase the risk of a Type II error.

### Strengths and limitations

An important strength of this study is that it represents a comprehensive, methodically sound RCT study, comparing the SoCoBo intervention with a highly appropriate control intervention (RehaCom^®^) and paying close attention to strict patient randomization. In addition, a comprehensive assessment battery, which made it possible to address not only the domain of social cognition, but also general cognition and variables associated with mental health, was implemented.

As a first limitation regarding the RCT conducted, it should be noted that the overall sample size remained relatively small for a comprehensive RCT study (with only 16 participants completing the control condition), albeit comparable to other RCTs published (for example Lissek et al. [76]). It would presumably have been possible to acquire a larger sample size, for example, by implementing a multi-center study. Also, to be able to evaluate long-term treatment effects, follow-up measurements weeks or months after the post assessment could have been included. Higher attrition rates to be expected with longer observation periods highlight the need for multicenter-studies ensuring higher sample sizes. Moreover, the implementation of another control group besides the control group including RehaCom^®^ would have allowed to provide more specific information on the efficacy of the SoCoBo-related effects, for example comparing SoCoBo to well-established programs that also focus on social cognition or to a conventional on-site treatment group. Furthermore, even though the localization of the TBI was assessed as part of the diagnostic process, it was not considered in the data analysis of this study because the localizations of the TBI were too multifaceted and diverse and the overall sample size was too small to obtain valid conclusions regarding specific localization patterns of the TBI relating to the results of this study (for an overview of the side the injury occurred see Results section). Moreover, the assessments were mostly based on self-reports and no assessment was carried out dealing with the possible generalization of the effects into everyday life due to the already very comprehensive assessments that were conducted in this study. Since in our study several dropouts exist, possible reasons for an early termination of the study can be discussed. Although this was not systematically recorded, as most dropouts were no longer available for giving a reason for dropping out of the study, it is possible that for some participants the total length of the intervention of 12 weeks and 4 sessions per week was too long to be motivated to participate until the end of the study. This highlights the need to design internet-based treatments that are as concise as possible or combined with on-site treatment to allow for more motivational support by a therapist. Other possible reasons might be problems in the personal environment, illness or significant changes to the life situation (e.g., job change) that made it impossible to continue the study. As another limitation of our study it should also be mentioned that the lack of blinding in this study may increase risk of bias affecting the results.

In general, when it comes to telerehabilitation, it should—even though several advantages of computerized approaches were presented in the introduction—also be mentioned at this point that computerized approaches can also be linked to several disadvantages, such as that appropriate technical equipment must exist for its use, that the transmission of sensitive personal information carries the risk of data breaches or unauthorized access or that some people might have difficulty using the computerized approaches due to age/inexperience, even though these disadvantages were not revealed to be an issue in the context of this study.

## Conclusion

Overall, it can be stated that the SoCoBo online therapy represents a promising new intervention tool for the amelioration of deficits in specific social cognition domains, as the results of this study indicate for the first time in a comprehensive RCT with TBI patients that it can improve (facial) emotion recognition ability as well as empathy. In the future, it would be important to evaluate whether the treatment effects only apply to patients with TBI, or whether patients with other etiologies could also benefit from the SoCoBo intervention. This would suggest that not only TBI patients benefit from SoCoBo, but that the findings found in this study can also be generalized to other diseases. Research on generalization of the effects into everyday life is especially urgent for the SoCoBo intervention as well as other computerized social cognition interventions in order to challenge the statement in the "INCOG 2.0 Guidelines for Cognitive Rehabilitation Following Traumatic Brain Injury, Part IV: Cognitive- Communication and Social Cognition Disorders" that at this stage of research computerized social cognition interventions are not recommended because of the lack of evidence of generalization to real life-activities [77]. When it comes to generalization, it would also be important to investigate whether the effects can be replicated in a similar way in other cultural contexts or populations. In addition, it would be interesting to find out in future studies what proportion of the intervention success can be attributed to therapeutic support, e.g., a study design could be implemented in which one group completes SoCoBo without any therapeutic support and another group with more extensive therapeutic support (e.g., regular telephone conversations as implemented in this study). In the long term SoCoBo is supposed to be an add-on treatment possibility complementing face-to-face therapies and allowing for a flexible and independent administration of the treatment modules (emotion recognition, perspective taking, social problem solving) as required based on the individual pattern of impairments observed in the patient. Also, to increase its effectiveness and promote generalization to everyday life SoCoBo psychoeducation and training should be complemented by individualized meaningful exercises based on the (social) activities of daily living that need to be carried out by the patient.

## Supporting information

S1 Checklist Reporting checklist for randomised trial. (DOCX)

S1 Appendix Number of the picture and video stimuli used in the practice sessions of the emotion recognition module, categorized by emotion (joy, disgust, fear, anger, sadness, surprise, shame, pride and jealous) and further characteristics. (DOCX)

S2 Appendix Characteristics of the audio plays (and written scenarios) used in the practice sessions of the perspective taking module, categorized by superordinate categories for the type of interaction. Numbers represent the number of audio plays per category.
(DOCX)

S3 Appendix Characteristics of the audio plays (and written scenarios) used in the practice sessions of the social problem-solving module, categorized by superordinate categories. Numbers represent the number of audio plays per category.
(DOCX)

S4 Appendix Description of the social cognition (a), general cognition (b) and clinical assessment tools (c).
(DOCX)

S5 Appendix Self-generated feedback questionnaire which was filled out by the participants after completing the respective program (SoCoBo vs. RehaCom®). (DOCX)

S6 Appendix Main effects and time*group interactions for the (social) cognition outcomes calculated for Hypotheses 1 and 2. (DOCX)

S7 Appendix Mean raw scores and standard deviation (SoCoBo and RehaCom®) for the self-generated feedback questionnaire items (answer options: 1–4; not at all, rather not, rather, strongly) with only those items presented that were provided for both programs. (DOCX)

S1 File (PDF)
