# Supplementary material for: Treating social cognition impairment with the online therapy ’SoCoBo’: A randomized controlled trial including traumatic brain injury patients
Source: PLoS One. 2024 Jan 10;19(1):e0294767. doi: 10.1371/journal.pone.0294767 (PMC10781160; doi:10.1371/journal.pone.0294767)
Supplement: S1 Appendix — (DOCX) [file pone.0294767.s002.docx]

| Characteristics | Joy | Disgust | Fear | Anger | Sadness | Surprise | Shame | Pride | Jealous | **Total** |
| --- | --- | --- | --- | --- | --- | --- | --- | --- | --- | --- |
| Static | 266 | 186 | 272 | 260 | 273 | 141 | 20 | 20 | 20 | **1458** |
| Dynamic | 119 | 74 | 119 | 119 | 120 | 73 | 26 | 26 | 26 | **702** |
| Face | 227 | 222 | 227 | 219 | 227 | 174 | 40 | 40 | 40 | **1416** |
| Body | 158 | 38 | 164 | 160 | 166 | 40 | 6 | 6 | 6 | **744** |
| Naturalistic | 340 | 260 | 346 | 334 | 348 | 214 | 46 | 46 | 46 | **1980** |
| Artificial | 45 | 0 | 45 | 45 | 45 | 0 | 0 | 0 | 0 | **180** |
| Male | 205 | 133 | 198 | 198 | 183 | 95 | 24 | 8 | 24 | **1068** |
| Female | 180 | 127 | 193 | 181 | 210 | 119 | 22 | 38 | 22 | **1092** |
| Young | 279 | 203 | 296 | 261 | 275 | 181 | 38 | 27 | 46 | **1606** |
| Old | 61 | 57 | 50 | 73 | 73 | 33 | 8 | 19 | 0 | **374** |
| Age not determinable | 45 | 0 | 45 | 45 | 45 | 0 | 0 | 0 | 0 | **180** |
| Single | 348 | 260 | 351 | 343 | 352 | 208 | 40 | 46 | 40 | **1994** |
| Interaction | 37 | 0 | 40 | 36 | 41 | 6 | 6 | 0 | 6 | **166** |
| **Total stimuli** | **385** | **260** | **391** | **379** | **393** | **214** | **46** | **46** | **46** | **2160** |

**S1 Appendix**

*Number of the picture and video stimuli used in the practice sessions of the emotion recognition module, categorized by emotion (joy, disgust, fear, anger, sadness, surprise, shame, pride and jealous) and further characteristics*
