# Supplementary material for: Treating social cognition impairment with the online therapy ’SoCoBo’: A randomized controlled trial including traumatic brain injury patients
Source: PLoS One. 2024 Jan 10;19(1):e0294767. doi: 10.1371/journal.pone.0294767 (PMC10781160; doi:10.1371/journal.pone.0294767)
Supplement: S2 Appendix — Numbers represent the number of audio plays per category. (DOCX) [file pone.0294767.s003.docx]

**S2 Appendix**

*Characteristics of the audio plays (and written scenarios) used in the practice sessions of the perspective taking module, categorized by superordinate categories for the type of interaction. Numbers represent the number of audio plays per category.*

|  | Joyful occasion | Faux pas | Compliment | Conflict | Misunder-standing | Surprise | **Total** |
| --- | --- | --- | --- | --- | --- | --- | --- |
| 1 speaker | 2 | 0 | 0 | 1 | 3 | 2 | **8** |
| 2 speakers | 12 | 13 | 11 | 16 | 17 | 12 | **81** |
| > 3 speakers | 4 | 7 | 7 | 9 | 6 | 3 | **36** |
| Length up to 1 minute | 5 | 2 | 1 | 0 | 2 | 0 | **10** |
| Length up to 2 minutes | 1 | 4 | 7 | 9 | 6 | 10 | **37** |
| Length up to 3 minutes | 6 | 6 | 6 | 5 | 6 | 1 | **30** |
| Length >3 minutes | 1 | 5 | 1 | 3 | 9 | 2 | **21** |
| Background sounds | 5 | 6 | 12 | 10 | 13 | 9 | **55** |
| No Background sounds | 8 | 11 | 3 | 7 | 10 | 4 | **43** |
| Written scenarios | 5 | 3 | 3 | 9 | 3 | 4 | **27** |
| Audio plays | 13 | 17 | 15 | 17 | 23 | 13 | **98** |
