# Supplementary material for: Treating social cognition impairment with the online therapy ’SoCoBo’: A randomized controlled trial including traumatic brain injury patients
Source: PLoS One. 2024 Jan 10;19(1):e0294767. doi: 10.1371/journal.pone.0294767 (PMC10781160; doi:10.1371/journal.pone.0294767)
Supplement: S3 Appendix — Numbers represent the number of audio plays per category. (DOCX) [file pone.0294767.s004.docx]

**S3 Appendix**

*Characteristics of the audio plays (and written scenarios) used in the practice sessions of the social problem-solving module, categorized by superordinate categories. Numbers represent the number*

*of audio plays per category.*

|  | Faux pas | Conflict | Misunder- standing | **Total** |
| --- | --- | --- | --- | --- |
| 1 speaker | 0 | 2 | 2 | **4** |
| 2 speakers | 21 | 14 | 30 | **65** |
| >3 speakers | 6 | 13 | 8 | **27** |
| Length up to 1 minute | 2 | 0 | 2 | **4** |
| Length up to 2 minutes | 3 | 9 | 8 | **20** |
| Length up to 3 minutes | 7 | 10 | 11 | **28** |
| Length >3 minutes | 8 | 3 | 10 | **21** |
| Background sounds | 6 | 10 | 16 | **32** |
| No Background sounds | 14 | 12 | 15 | **41** |
| Written scenarios | 7 | 7 | 9 | **23** |
| Audio plays | 20 | 22 | 31 | **73** |
