# Supplementary material for: Treating social cognition impairment with the online therapy ’SoCoBo’: A randomized controlled trial including traumatic brain injury patients
Source: PLoS One. 2024 Jan 10;19(1):e0294767. doi: 10.1371/journal.pone.0294767 (PMC10781160; doi:10.1371/journal.pone.0294767)
Supplement: S4 Appendix — Description of the social cognition (a), general cognition (b) and clinical assessment tools (c). (DOCX) [file pone.0294767.s005.docx]

**S4 Appendix**

*Description of the social cognition (a), general cognition (b) and clinical assessment tools (c)*

**a) Social Cognition**

Emotion Recognition Index (ERI; Scherer & Scherer^50^) & Geneva Emotion Recognition Test-Short form (GERT-S; Schlegel & Scherer^51^)

While the GERT-S is based on 42 short video clips including sounds, with ten different actors portraying 14 types of emotions, the ERI consists of a face subtest and a voice subtest, with the face subtest involving emotion expression stimuli that are presented for three seconds and the voice subtest containing pseudolinguistic sentences that are presented in the tone of a particular emotion. In both ERI and GERT, after each stimulus presentation, participants have to choose which emotion is portrayed by the actors in a forced-choice response format out of 5 (ERI) or 14 (GERT) alternatives. In both the ERI and the GERT, a percentage of correct answers is ultimately determined. While a Cronbach's alpha of .80 is reported for the GERT, a Cronbach's alpha was not calculated for the ERI, but the authors state that satisfactory construct validity could be achieved for the ERI by comparing the ERI with other measures that assess emotion recognition.

Saarbrücker Personality Questionnaire (German version of the Interpersonal Reactivity Index – IRI^52^)

The IRI is a 16-item questionnaire that assesses four different subfacets of empathy: Perspective taking (PT) and fantasy (FS) being related to cognitive empathy as well as empathic concern (EC) and personal distress (PD) being related to affective empathy. The perspective taking scale (PT) measures the ability to (spontaneously) perceive something from another person's perspective. The fantasy scale (FS) assesses - based on characters in novels or films - the tendency to understand the emotional world of these characters. The empathic concern scale (EC) was designed to measure other-oriented feelings such as concern or compassion for other people. The personal distress (PD) scale measures self-focused feelings in interpersonal situations (e.g., restlessness or discomfort). For the IRI, a total score is calculated (usually and also in this study involving the subscales fantasy, perspective taking and empathic concern). The score ranges from 12 to 60, with a higher value indicating greater empathic ability. The response format is a 5-point Likert scale (1 = never; 5 = always). A Cronbach's alpha between α = .63-.77 for the different subscales could be demonstrated for the German version of the IRI.

Inventory for Social Competencies - Short form (ISK-K^53^)

The ISK-K is a 33-item questionnaire assessing different aspects of social competencies. The ISK-K is composed of four different subscales: offensiveness, reflexivity, self-control, and social orientation. While the offensiveness scale (8 items) covers domains such as assertiveness, decisiveness, extraversion, and willingness to engage in conflicts, the reflexivity scale (7 items) deals with domains such as direct and indirect self-attention and self-presentation. The self-control scale (8 items) covers not only self-control, but also emotional stability or flexibility of action, and the social orientation scale (10 items) includes, among other things, items constructed to assess perspective taking, prosociality, and willingness to compromise. The score range is 8 to 32 for the offensiveness subscale, 7 to 28 for the reflexibility subscale, 8 to 32 for the self-control subscale and 10 to 40 for the social orientation subscale, with higher scores indicating greater social competence. The response format of the ISK-K is a 4-point Likert scale (1 = strongly disagree; 4 = strongly agree). Internal consistencies of > α =.55 for the different subscales were revealed. For the ISK, also a proxy assessment exists which was sent to relatives of the participants in this study.

Social Cognition Test Battery^54^

The Social Cognition Test Battery by Channon and Crawford contains a series of short stories depicting everyday, mostly interpersonal situations, which the participants are asked to read without a time limit. Different questions are then asked aimed at assessing how well certain social situations are understood and to what extent socially appropriate solutions to social problem situations are found. Both the free generation and the recognition of socially appropriate solutions out of a series of alternatives is assessed. The presentation of control questions ensures that the participants have understood the story with the test administrator being instructed to provide hints if a wrong answer is given, ensuring that socially inadequate answers are not due to a lack of understanding of the text. The Social Cognition Test Battery consists of three different subtests: The Mentalistic Interpretation Task (Part M) assessing ToM and the Social Problem Resolution Task (Part B: The best solution for each problem is supposed to be named) as well as the Social Problem Fluency Task (Part F – Fluency: As many good solutions as possible are supposed to be named within one minute) assessing social problem-solving in a performance-based manner. In part M, the participants were asked to verbally explain why the main character performed an action or why an event had happened or what the main character meant by a given remark. The interpretation quality of the explanations was assessed using a scoring system. Two points were awarded for answers that provided a clear correct explanation. One point was awarded for answers that were not wrong but inappropriate. Answers that were wrong and inappropriate were awarded zero points. Following the verbal explanation, the participants were presented with four alternative interpretations (one correct, one incorrect and two interpretations that were not necessarily incorrect but either irrelevant or much more general than the correct interpretation). Two points were awarded for choosing the correct interpretation, one point for choosing a correct but irrelevant or more general interpretation. Zero points were given for choosing the wrong interpretation. In relation to part B, the participants were asked to determine what would be the best thing for the main character to do in the situation described in the story. The solution quality of the answers was evaluated using criteria of social sensitivity and practical effectiveness. Solutions that were both socially sensitive and practically effective (SP) were awarded two points. One point was awarded for responses that were either socially sensitive but not practically effective (S) or practically effective but not socially sensitive (P). For inappropriate or irrelevant answers (N), zero points were given. With regard to part F it should first be answered why the situation described in the story might be awkward for the main character. If awkward elements of the situation were described a point was given, and no point was given if not. Then the participants were asked to find “good ideas” for solving the problem described in the story, with both socially sensitive and practically effective strategies being awarded two points, only socially sensitive or only practically effective strategies one point and responses that did not meet any criterion zero points (a maximum of 7 points could be reached at this point). The participants were then presented four different possible actions and they were asked to rank them in order from best to worst. Only if a socially sensitive and practically effective alternative was selected, the patients were rewarded two points. In this study, the Social Cognition Test Battery was divided into a version A and a version B, with the participants receiving a different version at the respective pre- and post-treatment assessments. At each assessment, 5 stories were presented from part B, 5 stories from part F and 8 (version A) or 7 (version B) stories from part M. For all subtests of the Social Cognition Test Battery (M, B, F), a total score was calculated based on the answers given, with possible scores ranging from 0 to 20 (part M), 0 to 10 (part B) and 0 to 50 (part F). For part M, a Cronbach's alpha of .62 (combined mentalistic items) is reported, whereas for part B, the reported alpha value is .58 and for part F it is α = .67.

Toronto Alexithymia Scale (TAS-20^55^)

The TAS-20 is a 20-item questionnaire, focusing on the deficiency in describing and identifying emotions. For the TAS-20, a three-factor structure was proposed: difficulty describing feelings (DDF), difficulty identifying feelings (DIF) and externally oriented thinking (EOT), the tendency to overlook one's own emotions. Scores range from 20 to 100, with a higher score indicating greater impairment. A 5-point Likert scale is used (1 = strongly disagree; 5 = strongly agree). In a validation study, a Cronbach's alpha of .81 was obtained for the TAS-20.

**b) General Cognition**

The Regensburg word fluency test (RWT^57^)

The RWT is a diagnostic test for assessing word fluency in which appropriate words have to be generated verbally over a period of one minute. The RWT contains subtests on formal lexical word fluency (naming as many words as possible for a specific letter [in this study: P/M] and semantic word fluency (naming as many terms as possible from a specific category [in this study: animals/groceries] or naming as many terms as possible switching back and forth between two categories [in this study: clothes and flowers/sports and fruit]).

Auditory Verbal Learning Test (AVLT^56^)

The AVLT is a neuropsychological test evaluating verbal memory. A list of 15 words is read out to the participant who is asked to remember as many words as possible. After five repetitions, a second list is presented (only one time) and the participant again is asked to remember as many words as possible. After this "interference trial" (and also after 20 minutes), the participant is again asked to name the words of the first list. Also, after 20 minutes, a list of 50 words is presented containing (among others) all of the words from the first and the second list and the participant has to state whether the words were included in the first list or not.

Wechsler Memory Scale: Digit span (Wechsler^58^)

The Digit Span being part of the Wechsler Memory Scale is a measure of working memory capacity. Participants hear a series of digits with the number of digits increasing after two trials each. Two variants of the Digit Span exist: The forward-span and the backward-span. In the forward-span variant, after hearing the complete list of digits the participants must recall the digits in the order they were presented. In the backward-span variant, after hearing the complete list of digits participants must recall the digits in the reverse order.

German version of the Stroop Test (FWIT^60^)

The Stroop test is a neuropsychological test that assesses information processing speed as well as resistance to interference. It consists of three subtests. In a first test, color words must be read as quickly as possible, and in a second test, colors must be named (again as quickly as possible). These first two subtests reflect information processing speed. In a third subtest, the color of a color word (that interferes with the color word itself) should be named (reflecting resistance to interference).

Scale for the Assessment of Action, Planning and Problem-solving Impairments (HPP-S^59^)

The HPP-S can be used to assess impairments in the planning and control of actions (executive dysfunction) after brain damage based on behavioral observations. The scale comprises 12 items with a 3-point Likert scale (0 = never true, 2 = often true). A total score can be calculated (range between 0 and 24), with higher scores indicating greater difficulties in the domain of executive functions. Cronbach's alpha values ranging between 0.82 to 0.84 could be determined.

**c) Clinical Assessment**

The Rasch-based Depression Screening (DESC^62^)

The DESC is a 10-item instrument used to assess depressive symptoms. The answers are five-point Likert-scaled ranging from never (0) to always (4). A total score can be calculated from the answers given (range = 0 to 40). The higher the total score, the greater the depressiveness of the participant. A total score ≥ 12 indicates the presence of a depressive episode. For the DESC, a Cronbach's alpha >. 90 could be determined.

The Social Interaction Anxiety Scale (SIAS^63^)

The SIAS is a questionnaire that measures stress when meeting and talking with other people containing 20 items. A 5-point Likert scale is used (0 = Not at all characteristic of me; 4 = Extremely characteristic of me), resulting in a total score range between 0 and 80, with higher values indicating greater stress. A Cronbach's alpha >. 90 could also be determined for the SIAS.

State-Trait Anxiety Inventory (STAI^64^)

The STAI is a questionnaire consisting of 40 items (4-point Likert scale with 1 = almost never; 4 = almost always) measuring two types of anxiety: state anxiety (20 items, form X1) and trait anxiety (20 items, form X2). A total score can be calculated for state anxiety and trait anxiety each (range = 20-80) with higher scores indicating higher levels of anxiety. Cronbach's alpha was high for the STAI form X1 as well as for the STAI form X2 (≥.90).

Questionnaire on Life Satisfaction (FLZ^65^)

The FLZ is used to assess relevant aspects of life satisfaction in different areas of life (e.g. health or occupation). The total of 70 items includes a seven-point Likert scale ranging from "very dissatisfied" (1) to "very satisfied" (7), resulting in a total score range from 70 to 490, with higher values indicating greater life satisfaction. Cronbach's alpha values for the FLZ are high and range between α = .82 and α = .95.
