# Supplementary material for: Treating social cognition impairment with the online therapy ’SoCoBo’: A randomized controlled trial including traumatic brain injury patients
Source: PLoS One. 2024 Jan 10;19(1):e0294767. doi: 10.1371/journal.pone.0294767 (PMC10781160; doi:10.1371/journal.pone.0294767)
Supplement: S5 Appendix — (DOCX) [file pone.0294767.s006.docx]

**S5 Appendix**

*Self-generated feedback questionnaire which was filled out by the participants after completing the respective program (SoCoBo vs. RehaCom®).*

Which device did you usually use for the program?

o Computer o Laptop o Tablet o Smartphone

Did you use the additional information materials and exercise sheets available?

o Yes o No

To what extent do the following statements apply to the program on a scale from "not at all" to "strongly"? (Answer options: Not at all, rather not, rather, strongly) – Statement 8 was not presented in the RehaCom® group, since in RehaCom® no psychoeducation sessions exist

1. I am satisfied with the program.

2. I am satisfied with the therapeutic support.

3. When using the program, I always knew what to do next.

4. The individual pages of the program were clearly laid out.

5. The individual pages of the training program were easy to read.

6. The navigation within the training program was intuitive.

7. The program as a whole was user-friendly.

8. The introductory explanations in the information sessions were understandable.

9. The tasks and instructions in the exercise sessions were understandable.

10. The daily scope of the program was adequate.

11. The overall duration of the program was appropriate.

12. I was motivated during the tasks.

13. Completing the training program was enjoyable for me.

14. The level of difficulty of the exercises was adequate.

15a. SoCoBo: Now, it is easier for me to recognize other people's feelings.

15b. RehaCom®: I have noticed improvements in terms of my memory.

16a. SoCoBo: Now, I am better able to put myself in other people’s thoughts and feelings.

16b. RehaCom®: I have noticed improvements in terms of my attention.

17a. SoCoBo: Now, I can solve problems more easily when talking to other people.

17b. RehaCom®: I have noticed improvements in terms of my executive functions.

18a. SoCoBo: The training content was useful to strengthen my social skills.

18b. RehaCom®: The content presented was useful to improve my mental abilities.

19. I was able to stick to the planed time schedule (4 sessions per week, 12 weeks in total).

Were there any technical difficulties while working on the tasks? If yes, please describe them.

__________________________________

What did you particularly like about this training program?

__________________________________

Do you have any specific suggestions on how to improve the training program?

Have you noticed any changes in your daily life or behavior during the last weeks since working with the training program?

__________________________________
