# Supplementary material for: Treating social cognition impairment with the online therapy ’SoCoBo’: A randomized controlled trial including traumatic brain injury patients
Source: PLoS One. 2024 Jan 10;19(1):e0294767. doi: 10.1371/journal.pone.0294767 (PMC10781160; doi:10.1371/journal.pone.0294767)
Supplement: S6 Appendix — (DOCX) [file pone.0294767.s007.docx]

**S6 Appendix**

*Main effects and time*group interactions for the (social) cognition outcomes calculated for Hypotheses 1 and 2*

| Outcomes Measures |  | |  | |  | |  |
| --- | --- | --- | --- | --- | --- | --- | --- |
|  |  |  |  | |  | |  |
|  | *F* | *df* | | *p* | | η^2^_p_ | |
| Social Cognition* |  |  | |  | |  | |
| Emotion recognition: ERI (faces); % correct |  |  | |  | |  | |
| Main Effect (Condition) | 2.116 | 1, 33 | | .155 | | .060 | |
| Time*Group Interaction | 4.245 | 1, 33 | | .047 | | .114 | |
| Emotion recognition: ERI (voices); % correct |  |  | |  | |  | |
| Main Effect (Condition) | .138 | 1, 33 | | .713 | | .004 | |
| Time*Group Interaction | .137 | 1, 33 | | .713 | | .004 | |
| Emotion recognition: GERT; % correct |  |  | |  | |  | |
| Main Effect (Condition) | 1.737 | 1, 32 | | .197 | | .051 | |
| Time*Group Interaction | 2.349 | 1, 32 | | .135 | | .068 | |
| Empathy: IRI Total (Perspective taking, Empathic concern, Fantasy) |  |  | |  | |  | |
| Main Effect (Condition) | .039 | 1,34 | | .844 | | .001 | |
| Time*Group Interaction | 4.359 | 1,34 | | .044 | | .114 | |
| Social Competencies: ISK-K (Social orientation, Offensiveness, Self-monitoring, Reflexibility) |  |  | |  | |  | |
| Main Effect (Condition) | .694 | 4, 26 | | .603 | | .096 | |
| Time*Group Interaction | .522 | 4, 26 | | .720 | | .074 | |
| ToM: SCAMPS-M Total |  |  | |  | |  | |
| Main Effect (Condition) | 1.343 | 1, 37 | | .254 | | .035 | |
| Time*Group Interaction | .041 | 1, 37 | | .840 | | .001 | |
| Social Problem Solving: SCAMPS-B Total |  |  | |  | |  | |
| Main Effect (Condition) | .124 | 1, 37 | | .727 | | .003 | |
| Time*Group Interaction | .881 | 1, 37 | | .354 | | .023 | |
| Social Problem Solving: SCAMPS-F Total |  |  | |  | |  | |
| Main Effect (Condition) | .826 | 1, 35 | | .370 | | .023 | |
| Time*Group Interaction | .577 | 1, 35 | | .452 | | .016 | |
| Alexithymia: TAS-20 Total |  |  | |  | |  | |
| Main Effect (Condition) | 3.571 | 1, 34 | | .067 | | .095 | |
| Time*Group Interaction | .111 | 1, 34 | | .741 | | .003 | |
| General Cognition |  |  | |  | |  | |
| Regensburg word fluency test (Formal lexical, Semantic, Semantic Change) |  |  | |  | |  | |
| Main Effect (Condition) | .630 | 3, 34 | | .601 | | .053 | |
| Time*Group Interaction | .196 | 3, 34 | | .898 | | .017 | |
| Digit Span Forwards / Backwards; seconds |  |  | |  | |  | |
| Main Effect (Condition) | .089 | 2, 36 | | .915 | | .005 | |
| Time*Group Interaction | .563 | 2, 36 | | .575 | | .030 | |
| Auditory Verbal Learning Test (AVLT 1-5, AVLT 5-6, AVLT 5-7) |  |  | |  | |  | |
| Main Effect (Condition) | .055 | 3, 35 | | .983 | | .005 | |
| Time*Group Interaction | .707 | 3, 35 | | .554 | | .057 | |
| Stroop Test (Color word reading, Color naming, Interference); seconds |  |  | |  | |  | |
| Main Effect (Condition) | 1.466 | 3, 35 | | .241 | | .112 | |
| Time*Group Interaction | .333 | 3, 35 | | .802 | | .028 | |
| HPP-S Total |  |  | |  | |  | |
| Main Effect (Condition) | 1.076 | 1, 33 | | .307 | | .032 | |
| Time*Group Interaction | .446 | 1, 33 | | .509 | | .013 | |

*Note.* ERI = Emotion Recognition Index; GERT = Geneva Emotion Recognition Test-Short form; HPP-S = the Scale for the assessment of action, planning and problem-solving impairments; IRI = Interpersonal Reactivity Index; ISK-K = Inventory for Social Competencies - Short form; SCAMPS = ToM and social problem solving tasks

* If not explicitly stated otherwise in the respective lines, the analyses are based on raw scores
