# Supplementary material for: Treating social cognition impairment with the online therapy ’SoCoBo’: A randomized controlled trial including traumatic brain injury patients
Source: PLoS One. 2024 Jan 10;19(1):e0294767. doi: 10.1371/journal.pone.0294767 (PMC10781160; doi:10.1371/journal.pone.0294767)
Supplement: S7 Appendix — (DOCX) [file pone.0294767.s008.docx]

**S7 Appendix**

*Mean raw scores and standard deviation (SoCoBo and RehaCom®) for the self-generated feedback questionnaire items (answer options: 1-4; Not at all, rather not, rather, strongly) with only those items presented that were provided for both programs*

|  | SoCoBo (*N* = 22) | RehaCom® (*N* = 14) |
| --- | --- | --- |
| I am satisfied with the program. | 3.41 (*SD* = .503) | 3.00 (*SD* = .679) |
| I am satisfied with the therapeutic support. | 3.59 (*SD* = .590) | 3.86 (*SD* = .363) |
| When using the program, I always knew what to do next. | 3.45 (*SD* = .596) | 3.36 (*SD* = .497) |
| The individual pages of the program were clearly laid out. | 3.59 (*SD* = .503) | 3.43 (*SD* = .514) |
| The individual pages of the training program were easy to read. | 3.59 (*SD* = .590) | 3.50 (*SD* = .519) |
| The navigation within the training program was intuitive. | 3.55 (*SD* = .510) | 3.43 (*SD* = .514) |
| The program as a whole was user-friendly. | 3.59 (*SD* = .734) | 3.36 (*SD* = .633) |
| The tasks and instructions in the exercise sessions were understandable. | 3.59 (*SD* = .503) | 3.43 (*SD* = .514) |
| The daily scope of the program was adequate. | 3.45 (*SD* = .596) | 3.43 (*SD* = .514) |
| The overall duration of the program was appropriate. | 3.55 (*SD* = .510) | 3.57 (SD = .514) |
| I was motivated during the tasks. | 3.45 (*SD* = .596) | 3.36 (*SD* = .745) |
| Completing the training program was enjoyable for me. | 3.45 (*SD* = .596) | 3.21 (*SD* = .802) |
| The level of difficulty of the exercises was adequate. | 3.27 (*SD* = .703) | 2.79 (*SD* = .802) |
| I was able to stick to the planed time schedule (4 sessions per week, 12 weeks in total). | 3.32 (*SD* = .716) | 3.43 (*SD* = .646) |
