# Supplementary material for: Treating social cognition impairment with the online therapy ’SoCoBo’: A randomized controlled trial including traumatic brain injury patients
Source: PLoS One. 2024 Jan 10;19(1):e0294767. doi: 10.1371/journal.pone.0294767 (PMC10781160; doi:10.1371/journal.pone.0294767)
Supplement: S1 File — (PDF) [file pone.0294767.s009.pdf]

Antrag zur Beurteilung eines Forschungsvorhabens an die Ethik-Kommission der Fakultät für Psychologie der Ruhr-Universität Bochum

Allgemeine Angaben

1. **Dem Antrag liegen bei:** *Bitte ankreuzen*

   x Einverständniserklärung

   x Probandenaufklärung

   x Tabellarischer Ablauf des Forschungsvorhabens

2. **Titel der Studie**

   Hier einfügen: *Entwicklung und Wirksamkeitsprüfung eines internetgestützten Therapieprogrammes zur Behandlung von Einschränkungen Sozialer Kognitionen und Kompetenzen nach erworbener Hirnschädigung*

3. **Kurze Zusammenfassung des Studienvorhabens (Ziele, Stichprobe (Versuchspersonenanzahl), methodisches Vorgehen; max. 250 Wörter)**

   Hier einfügen: Das wichtigste Ziel unseres Vorhabens besteht darin, ein internetgestütztes Programm zur Behandlung von Einschränkungen der Sozialen Kognitionen und Kompetenzen nach Hirnschädigung zu entwickeln. Probleme in diesen Bereichen haben gravierende Konsequenzen für die interpersonellen Beziehungen, die berufliche Wiedereingliederung und die gesellschaftliche Teilhabe hirngeschädigter Personen, werden aber häufig aufgrund mangelnder Behandlungsansätze und einer Unterversorgung im Bereich der ambulanten Neuropsychologie kaum berücksichtigt. Das neue Programm soll in drei Modulen die Fähigkeiten, die Gefühle anderer Menschen anhand von Informationen in Gesichtern und Körperhaltungen zu erkennen (Emotionserkennung), die Gedanken und Gefühle Anderer aus dem Situationskontext zu erschließen (kognitive und emotionale Perspektivübernahme) und sich, basierend auf den vorgenannten Fähigkeiten, in schwierigen interpersonellen Situationen angemessen zu verhalten (Soziales Problemlösen), verbessern. Jedes Modul bietet internetgestützt einen Psychoedukationsteil und einen praktischen Übungsteil. In der anschließenden Evaluation soll die Wirksamkeit des Programms im Hinblick auf die Rehabilitation soziokognitiver Einschränkungen anhand einer 16 Wochen dauernden Therapie überprüft werden. Dazu soll eine randomisierte kontrollierte Studie mit zwei Gruppen von Personen nach Schädel-Hirn-Traumata oder Schlaganfällen (je N = 30), die Defizite im Bereich der Sozialen Kognitionen aufweisen, durchgeführt werden: Die Experimentalgruppe wird mit dem neuen Programm im Hinblick auf Emotionserkennung, Perspektivübernahme und Soziales Problemlösen behandelt, während die Kontrollgruppe im gleichen Zeitraum mit gleicher Intensität (5 x pro Woche, 30-60 Minuten) ein ebenfalls internetgestütztes Programm zur Behandlung von Defiziten in den kognitiven Bereichen Alertness, Arbeitsgedächtnis und exekutive Funktion absolviert. Begleitet wird die Therapie durch 14tägig erfolgende therapeutische Telefonkontakte. Eine umfassende Prä-Post-Diagnostik soll klären, inwiefern unser Programm eine spezifische und signifikante Verbesserung soziokognitiver Leistungen bewirkt.

4. **Name und Anschrift der/ des verantwortlichen Forscherin / Forschers**

   Name, Vorname: *Hier einfügen:* PD Dr. Thoma, Patrizia

   Anschrift: *Hier einfügen:* Neuropsychologisches Therapie Centrum (NTC), AG Klinische Neuropsychologie, Fakultät für Psychologie, Ruhr-Universität Bochum

Telefonnr.: *Hier einfügen:* [redacted]

E-Mail: *Hier einfügen:* [redacted]

5. **Sind weitere Forscherinnen / Forscher der Fakultät für Psychologie an dem Forschungsvorhaben beteiligt?**

   Namen: *Hier einfügen:* Prof. Dr. Boris Suchan

6. **Erstmalige Beantragung: Wurde der Antrag bereits bei einer anderen Ethikkommission zur Begutachtung eingereicht?** *Bitte ankreuzen*

   ☐ Ja

   X Nein

[redacted]

Bochum, den 13.12.2017

Ort, Datum	Unterschrift der durchführenden Forscherin bzw. des durchführenden Forschers
Ort, Datum	(Ggf.) Unterschrift der Betreuerin bzw. des Betreuers

Checkliste zur Studie:

	ja	nein
**1. Freiwilligkeit:**<br>Ist die Freiwilligkeit der Teilnahme gewährleistet?	x	☐
**2. Geschäftsfähigkeit:**<br>Werden an der Studie Personen teilnehmen, die nicht selbst ihre Zustimmung zur Teilnahme geben können (z.B. Personen unter 18 Jahren, Personen, die nicht im juristischen Sinne einwilligungsfähig sind)?	☐	x
**3. Beeinträchtigte Personengruppe:**<br>Werden an der Studie Personen teilnehmen, die einer besonders verletzlichen Gruppe angehören (z.B. klinische Stichproben, Personen mit Lernschwäche, Personen im Klinik- oder Strafvollzugssetting)?	x	☐
**4. Ein- und Ausschlusskriterien:**<br>Gibt es für die Probanden Ein- und/ oder Ausschlusskriterien?	x	☐
**5. Täuschung über Teilnahme:**<br>Ist es erforderlich, dass Personen an der Studie teilnehmen, ohne zu diesem Zeitpunkt über ihre Teilnahme informiert zu sein bzw. ohne ihre Einwilligung gegeben zu haben (z.B. bei nicht-offener Beobachtung) oder dass sie nicht vollständig über Zweck und Inhalt der Studie informiert werden (Anm.: die Offenlegung der Hypothesen zählt nicht hierzu)?	☐	x
**6. Täuschung über Zweck:**<br>Werden Personen aktiv über den Inhalt und den Zweck der Studie getäuscht?	☐	x
**7. Intimität/ Stigmatisierung:**<br>Werden Fragen zu Themen gestellt, die für die Befragten von intimer Natur sind oder deren Beantwortung als stigmatisierend wahrgenommen werden kann (z.B. zu illegalem oder deviantem Verhalten)?	☐	x
**8. Belastung:**<br>Ist zu erwarten, dass die Teilnehmer/innen durch die Studie psychischen Stress, Furcht, Erschöpfung, Schmerzen oder andere negative Effekte erleiden, die über das im Alltag zu erwartende Maß hinausgehen?	☐	x
**9. Risiken:**<br>Werden die Teilnehmer/innen der Studie irgendwelchen invasiven oder potenziell schädlichen Prozeduren unterzogen?	☐	x
**10. Substanzvergabe:**<br>Werden den Teilnehmer/innen in der Studie Medikamente, Placebos oder andere Substanzen verabreicht?	☐	x
**Achtung:**<br>**Die folgenden Fragen 11-13 müssen nur beantwortet werden, wenn personenbezogene Daten erhoben werden. Falls keine personenbezogenen Daten erhoben werden, gehen Sie direkt über zu Frage 14.**		
**11. Datenschutz:**<br>Die Datensicherheit der personenbezogenen Daten ist entsprechend dem angefügten Merkblatt zur Datensicherheit (siehe Anlage) gewährleistet.	x	☐
**12. Datenschutzinformation:**<br>Probanden werden über die Datensicherheit der personenbezogenen Daten informiert.	x	☐
**13. Recht auf Datenlöschung:**<br>Die Probanden können jederzeit die Löschung/Vernichtung Ihrer personenbezogenen Daten verlangen und werden darüber informiert?	x	☐
**14. Versicherungsschutz:**<br>Besteht für die Probanden eine Wegeversicherung oder werden die Probanden darüber aufgeklärt, dass der Anfahrtsweg nicht versichert ist? (Anm.: Falls eine Wegeversicherung besteht, sollte die Police einsehbar im Sekretariat hinterlegt sein)	x	☐

Anmerkung:
Genauere Informationen zu einzelnen Themen können der folgenden Internetseite entnommen werden: https://www.dgps.de/index.php?id=186
Wenn Sie eine oder mehrere der Fragen in den grau unterlegten Anwortfeldern angekreuzt haben (d.h., Fragen 1, bzw. 11- 14 mit „Nein“ oder eine oder mehrere Fragen 2-10 der Checkliste mit "ja"

beantwortet haben), stellen Sie bitte in dem beigefügten Blatt kurz und präzise die Notwendigkeit des/r Punkte(s) dar. Gehen Sie ebenfalls darauf ein, wie Sie dafür Sorge tragen werden, dass in Hinsicht auf diese(n) Punkt(e) die Ethikrichtlinien eingehalten werden. Falls Sie mehr Platz benötigen, fügen Sie dem Antrag bitte ein separates Dokument hinzu.

Bitte beachten Sie, dass es in jedem Fall erforderlich ist, Teilnehmer/innen vorab so detailliert wie möglich über den Ablauf einer Studie zu informieren, ihnen mitzuteilen, dass sie jederzeit freiwillig die Studie abbrechen können, ihre informierte Einwilligung schriftlich einzuholen und Vertraulichkeit der Datenerhebung und –speicherung zu gewährleisten. Sollten sich im Verlauf der Erhebung wesentliche Änderungen der Studie ergeben, ist die Ethikkommission erneut zu konsultieren.

Ich bestätige, dass alle Angaben in diesem Fragebogen nach bestem Wissen zutreffend sind.

[redacted]

Bochum, den 13.12.2017

Ort, Datum

Unterschrift der durchführenden Forscherin bzw. des durchführenden Forschers

Ort, Datum

(Ggf.) Unterschrift der Betreuerin bzw. des Betreuers

Anmerkungen zur Checkliste:

Hier einfügen:

Zu 3.) Da es in der Studie um die Wirksamkeitsprüfung für ein neu zu entwickelndes Programm zur Behandlung soziokognitiver Einschränkungen nach erworbenen Hirnschädigungen geht, ist die Untersuchung hirngeschädigter Personen unumgänglich.

Zu 4.) Folgende Ein- und Ausschlusskriterien werden zugrunde gelegt:

Einschlusskriterien:

- Diagnose eines Schädel-Hirn-Traumas oder eines Schlaganfalls in den vergangenen fünf Jahren
- Altersbereich zwischen 18 und 55 Jahren
- geschätzter verbaler Intelligenzquotient über 80
- ausreichend gute Kenntnisse der deutschen Sprache
- Einschränkungen im Bereich der Sozialen Kognitionen und Kompetenzen, erfasst über beeinträchtigte Werte im Selbst- und Fremdbericht im „Inventar Sozialer Kompetenzen“ nach Kanning et al. (2009), möglichst gestützt durch Berichte über das Vorliegen soziokognitiver Defizite aus aktuellen oder Vorbehandlungen
- postakute (ambulante) Rehabilitationsphase
- Basiskenntnisse im Umgang mit PCs; ggf. Unterstützung durch versierte Angehörige; internetfähiger PC und E-Mail-Konto vorhanden

Ausschlusskriterien:

- Aktuelle Erfüllung von Diagnosekriterien für Erkrankungen aus dem Psychosespektrum/ für Abhängigkeitserkrankungen. Die häufig mit erworbenen

Hirnschädigungen einhergehenden Depressionen, Anpassungs- und Angststörungen werden als Komorbiditäten zugelassen.

- progrediente Erkrankungen des Zentralen Nervensystems (z.B. Demenzen)
- mittel bis schwer ausgeprägte Aphasien, ein amnestisches Syndrom, schwere Ausprägungen von Gesichtsfeldeinschränkungen oder Neglect, schwere motorische Beeinträchtigungen im Hand-/Arm Bereich
- bereits erfolgte Teilnahme an Therapien zur Verbesserung soziokognitiver Kompetenzen (z.B. Gruppentraining Sozialer Kompetenzen)

Diagnostik: Prä-Post-Untersuchungen

Tabelle 1: Testbatterie zur neuropsychologischen Eingangs- und Abschlussdiagnostik

**SOZIALE KOGNITIONEN**	**Dauer**
o Inventar Sozialer Kompetenzen im Selbst- und Fremdbild (Kanning, 2009)	5 Min
o Social Phobia Scale und Social Interaction Anxiety Scale (Mattick und Clarke, 1999; dt. Version von Stangier et al., 1998)	5 Min
o Empathie: Saarbrücker Persönlichkeitsfragebogen (Paulus, 2012)	5 Min
o Toronto Alexithymie Skala 20 (Taylor et al., 1985; dt. Version von Kupfer et al., 2001)	5 Min
o Tübinger Affektbatterie, Subtests zur Emotionserkennung in Gesichtern (Breitenstein et al., 1996)	15 Min
o Kognitive und Emotionale Perspektivübernahme: Movie for the Assessment of Social Cognition (MASC) (Dziobek et al., 2006)	20 Min
o Soziale Problemlösekompetenzen: SCAMPS (Channon und Crawford, 2010)	
Test zur Untersuchung Sozialen Problemlösens: Teile *M* (Perspektivübernahme in problematischen sozialen Interaktionssituationen), *F* (Generierung so vieler Lösungen wie möglich für schwierige interpersonelle Situationen) und *B* (Generierung optimaler Lösungsstrategien). Alle Teile liegen in zwei von uns entwickelten deutschsprachigen gegenüber dem englischen Original verkürzten Parallelversionen vor (hier verwendet: Thoma et al., 2015; Schmidt et al., 2016)	25 Min
**EXEKUTIVE FUNKTIONEN**	
o Kognitive Flexibilität (Testbatterie zur Aufmerksamkeitsprüfung: Zimmermann und Fimm, 2002)	5 Min
o Planungstest (Kohler et al., 2003)	15 Min
o GoNogo (Testbatterie zur Aufmerksamkeitsprüfung: Zimmermann und Fimm, 2002)	5 Min
o Regensburger Wortflüssigkeitstest (Aschenbrenner et al., 2001)	5 Min
**GEDÄCHTNIS**	
o Zahlen- und Blockspanne aus der Wechsler Gedächtnisskala (Wechsler, 2012)	10 Min
o Verbaler Lern- und Merkfähigkeitstest (Helmstaedter et al., 2001)	10 Min
o Rey-Osterrieth Figur (Osterrieth, 1944)	5 Min
**AUFMERKSAMKEIT**	
o Testbatterie zur Aufmerksamkeitsprüfung (Zimmermann & Fimm, 2002)	
o Alertness tonisch und phasisch	5 Min
o Geteilte Aufmerksamkeit (Version „Töne und Quadrate" oder „Töne und Zahlen")	5 Min
**KLINISCHE SKALEN UND INTELLIGENZ**	
o BDI II (Beck et al., 2009, dt. Version von Hautzinger et al., 2009)	5 Min
o State -Trait Angstinventar (Laux et al., 1981)	5 Min
o Persönlichkeitsstil- und Störungsinventar (Kuhl und Kanzen, 2009)	10 Min
o Prämorbide Intelligenz (nur bei Eingangsdiagnostik): Mehrfachwahl-Wortschatztest (Lehrl, 2005)	5 Min
o Fragebogen zur Lebenszufriedenheit (Fahrenberg et al., 2000)	5 Min
o Mini DIPS Diagnostisches Kurzinterview bei psychischen Störungen (nur bei Eingangsdiagnostik) (Margraf, 1994)	30 Min

Die Untersuchungen der Prä- und Postdiagnostik (vor und nach 16 Wochen Therapie, s.u.) werden jeweils auf 2 Sitzungen verteilt, um die Belastung für die Patienten möglichst gering zu halten.

Therapeutisches Rational:

Es werden zwei Gruppen von Patientinnen und Patienten 16 Wochen lang behandelt: Die Experimentalgruppe absolviert das neue „Programm zur Behandlung Sozialer Kognitionen und Kompetenzen der Ruhr-Universität Bochum“ – („SoKoBo“), die Kontrollgruppe mit gleicher Trainingsintensität ein ebenfalls internetgestütztes Trainingsprogramm („Rehacom“ der Firma Hasomed, Magdeburg), welches auf die Verbesserung kognitiver Einschränkungen ausgerichtet ist.

Bei „SoKoBo“ wird es sich um ein therapeutisch begleitetes internetgestütztes Programm handeln, welches drei Module umfasst, wobei jedes einen Psychoedukationsteil und einen praktischen Teil beinhalten wird. Im Modul „Emotionserkennung“ soll das Erkennen von Emotionen in Gesichtern und Körpern trainiert werden. Im Modul „Kognitive und Emotionale Perspektivübernahme“ soll anhand von Filmsequenzen das Einnehmen der gedanklichen und gefühlsmäßigen Perspektive anderer Personen geübt werden. Im Modul „Soziales Problemlösen“ soll schließlich, ebenfalls anhand von Filmsequenzen das Entwickeln sozial verträglichen und gleichzeitig praktisch effektiven Lösungsstrategien für schwierige soziale Situationen trainiert werden.

Die täglichen Therapieeinheiten (fünf Mal pro Woche, 30-60 Minuten lang) werden für die Teilnehmenden anhand des Studienprotokolls internetgestützt vom therapeutischen Personal voreingestellt. Dieses kann einsehen, inwiefern die Übungseinheiten bearbeitet wurden und so ggf. Therapielücken im Rahmen der vierzehntägig erfolgenden Telefongespräche (ca. 50 Minuten) thematisieren. Dabei wird strukturiert nach Fortschritten und Problemen beim Absolvieren der Übungspakete, beim Durchführen der „Hausaufgaben“ im Alltag sowie nach dem allgemeinen Befinden gefragt. Außerdem werden Rückmeldungen zu den erreichten Leistungen gegeben.

In der Experimentalgruppe bauen die drei Therapiemodule konsekutiv aufeinander auf. Zu Beginn steht ein neu eingeführtes Modul jeweils mit hoher Behandlungsfrequenz im Vordergrund, anschließend werden die Einheiten der drei Therapiemodule zunehmend gleichmäßig über den gesamten Behandlungszeitraum verteilt (siehe Tabelle 2), um eine Stabilisierung und Synergieeffekte der Therapieerfolge zu erreichen. Die Psychoedukations- und Übungseinheiten werden dabei jeweils miteinander verzahnt. Das Modul „Emotionserkennung“ soll als basaler und weniger komplex zu vermittelnder Therapiebaustein insgesamt 20, die Module „Perspektivübernahme“ und „Soziales Problemlösen“ sollen jeweils 30 Trainingstage umfassen. In der Kontrollgruppe werden mit gleicher Verteilung drei nach SHT häufig beeinträchtigte Funktionsbereiche trainiert: Basale Aufmerksamkeitsfunktionen (hinsichtlich der Frequenz analog zur Emotionserkennung), Arbeitsgedächtnis und Handlungsplanung (analog zu Perspektivübernahme und Sozialem Problemlösen). Diesen Funktionsbereichen werden aus dem Rehacom-Programm die Aufgaben „Alertnesstraining“, „Arbeitsgedächtnis“ und „Einkauf“ zugeordnet.

Im Rahmen der Studie durchlaufen zwecks Vergleichbarkeit alle Teilnehmenden das Programm nach einem standardisierten Protokoll, das eine gleichmäßige Applikation der Module in gleicher Trainingsintensität vorsieht. Die praktische Anwendung wird

auch eine individualisierte Handhabung ermöglichen (z.B. Weglassen einzelner Module; leistungsadaptive Veränderung der Schwierigkeitsstufen).

Tabelle 2: Übersichtsplan Therapieverlauf

**Woche**	**1**	**2 (T)**	**3**	**4 (T)**	**5**	**6 (T)**	**7**	**8 (T)**	**9**	**10 (T)**	**11**	**12 (T)**	**13**	**14 (T)**	**15**	**16 (T)**
**EE, AT**																
**PÜ, AG**																
**SP, EK**																

**LEGENDE**			
	5 Tage die Woche	*Module in Experimentalgruppe*	*Module in Kontrollgruppe*
	2 Tage die Woche	EE: Emotionserkennung	AT: Alertnesstraining
	1 Tag die Woche	PÜ: Perspektivübernahme	AG: Arbeitsgedächtnis
**(T)**	Therapeutischer Telefonkontakt	SP: Soziales Problemlösen	EK: Einkauf

English translation

Title of the study:

Development and efficacy evaluation of an internet-based therapy program for the treatment of impairments in social cognition and social skills after acquired brain injury

Brief summary of the study:

The main goal of our project is to develop an internet-based program for the treatment of impairments in social cognition and skills after brain injury. Problems in these domains have serious consequences for interpersonal relationships, occupational reintegration and social participation of brain-injured individuals but are often poorly addressed due to a lack of treatment approaches in the field of outpatient neuropsychology. Including three modules, the new program aims to improve the ability to recognize other people's feelings based on information in faces and body postures (emotion recognition), to infer the thoughts and feelings of others from the context of the situation (cognitive and emotional perspective taking) and, based on the aforementioned skills, to behave appropriately in difficult interpersonal situations (social problem solving). Each module offers an internet-based psychoeducation part and a practical exercise part. In the subsequent evaluation study, the effectiveness of the program with regard to the rehabilitation of socio-cognitive impairments is to be tested on the basis of a 16-week intervention. For this purpose, a randomized controlled trial will be conducted with two groups of subjects (traumatic brain injury or stroke, N = 30 each) who show deficits in the area of social cognition. The experimental group is treated with the new program with regard to emotion recognition, perspective taking and social problem solving, while the control group completes an Internet-based program for the treatment of deficits in the cognitive areas of alertness, working memory and executive function with the same intensity (5 sessions per week, 30-60 minutes) during the same period. The therapy is accompanied by therapeutic telephone contacts every 14 days. Comprehensive pre-post assessments will clarify to what extent the program leads to a specific and significant improvement in socio-cognitive performance.

Inclusion criteria:

- Diagnosis of a traumatic brain injury or stroke in the past five years
- Age range between 18 and 55 years
- Verbal intelligence quotient above 80
- Sufficiently good knowledge of the German language
- Impairments in the area of social cognition and competencies, recorded via impaired values in the self- and third-party report in the "Inventory of Social Competences" according to Kanning et al. (2009), if possible supported by reports on the presence of socio-cognitive deficits from current or pre-treatments
- Post-acute (outpatient) rehabilitation phase
- Basic knowledge in the use of PCs; if necessary, support by experienced relatives; Internet-capable PC and e-mail account available

Exclusion criteria:

- Current fulfilment of diagnostic criteria for disorders from the psychosis spectrum/ for dependency disorders. Depression, adjustment and anxiety disorders, which often accompany acquired brain injuries, are admitted as comorbidities
- Progressive diseases of the central nervous system (e.g. dementias)
- Moderate to severe aphasia, an amnesic syndrome, severe manifestations of visual field impairment or neglect, severe motor impairments in the hand/arm
- Previous participation in therapies for the improvement of socio-cognitive skills (e.g. group therapy)

Pre-post assessments:

Social Cognition

- The Inventory for Social Competencies - Short form (ISK-K), Kanning, 2009 (5 Min)
- Social Phobia Scale und Social Interaction Anxiety Scale, Mattick und Clarke, 1999 (5 Min)
- German version of the Interpersonal Reactivity Index (IRI), Paulus, 2012 (5 Min)
- The Toronto Alexithymia Scale (TAS-20), Taylor et al., 1985 (5 Min)
- Tübingen Affect Battery, subtests for emotion recognition in faces, Breitenstein et al., 1996 (15 Min)
- Movie for the Assessment of Social Cognition (MASC), Dziobek et al., 2006 (20 Min)

- SCAMPS, Channon and Crawford, 2010 (25 Min)

General Cognition

- Tests for Attentional Performance, Zimmermann und Fimm, 2002 (20 Min)
- Planning test, Kohler et al., 2003 (15 Min)
- The Regensburg word fluency test (RWT), Aschenbrenner et al., 2001 (5 Min)
- Digit span, Wechsler, 2012 (10 Min)
- Auditory Verbal Learning Test (AVLT), Helmstaedter et al., 2001 (10 Min)
- Rey-Osterrieth complex figure (ROCF), Osterrieth, 1944 (5 Min)

Clinical Scales and Intelligence

- Beck Depression Inventory II, Beck et al., 2009 (5 Min)
- State-Trait Anxiety Inventory (STAI), Laux et al., 1981 (5 Min)
- Personality Style and Disorder Inventory, Kuhl and Kazen, 2009 (10 Min)
- German vocabulary intelligence test (MWT-B, only pre-assessment), Lehrl, 2005 (5 Min)
- Questionnaire on Life Satisfaction (FLZ), Fahrenberg et al., 2000 (5 Min)
- Mini-DIPS: Diagnostic Short-Interview for Mental Disorders (only pre-assessment), Margraf, 1994 (30 Min)

The pre- and post-assessments (before and after 16 weeks of therapy, see below) are each spread over 2 sessions to minimize the burden on patients.

Therapeutic rationale:

Two groups of patients are treated for 16 weeks: The experimental group completes the new "Program for the Treatment of Social Cognitions and Competencies of the Ruhr University Bochum" - ("SoCoBo"), the control group with the same training intensity completes a likewise internet-based training program ("Rehacom" of the company Hasomed, Magdeburg), which is aimed at improving cognitive impairments. SoCoBo will be a therapeutically accompanied internet-based program comprising three modules, each of which will include a psychoeducation part and a practical part. In the module "Emotion Recognition", the recognition of emotions in faces and bodies will be trained. In the module "Cognitive and Emotional Perspective Taking", the assumption of the mental and emotional perspective of other persons is to be practiced on the basis of film sequences. Finally, in the module "Social Problem Solving", the development of socially acceptable and at the same time practically effective solution strategies for difficult social situations is to be trained, also with the help of film sequences. The daily therapy sessions (five times a week, 30-60 minutes each) are preset for

the participants by the therapeutic staff. The therapeutic staff can see to what extent the exercise units have been worked on and, if necessary, address therapy gaps during the fortnightly telephone conversations (approx. 50 minutes). In this context, structured questions are asked about progress and problems in completing the exercise packages, in carrying out the "homework" in everyday life as well as about the general state of health. In addition, feedback is given on the achievements. In the experimental group, the three therapy modules build on each other consecutively. The units of the three therapy modules are evenly distributed over the entire treatment period with high treatment frequency at the beginning and lowered frequency in the course of the training (see Overview below) in order to achieve stabilization and synergy effects of the therapy successes. The psychoeducation and exercise units are each linked together. The module "Emotion Recognition", as a basic and less complex therapy component, should comprise a total of 20 training days, the modules "Perspective Taking" and "Social Problem Solving" should each comprise 30 training days. In the control group, three functional areas frequently impaired after TBI are trained: Basic attention functions (analogous to emotion recognition in terms of frequency), working memory and action planning (analogous to perspective taking and social problem solving). The tasks "alertness training", "working memory" and "shopping" from the Rehacom program are assigned to these functional areas. For the purpose of comparability, all study participants complete the program according to a standardized protocol that provides for uniform application of the modules with the same training intensity. The practical application will also allow for individualized handling (e.g. omission of individual modules; performance-adaptive change of difficulty levels).

Overview of the course of the online therapy

**Week**	**1**	**2 (P)**	**3**	**4 (P)**	**5**	**6 (P)**	**7**	**8 (P)**	**9**	**10 (P)**	**11**	**12 (P)**	**13**	**14 (P)**	**15**	**16 (P)**
**ER, AT**																
**PT, WM**																
**SP, SH**																

**Key**			
	5 days per week	*Modules in experimental group*	*Modules in control group*
	2 days per week	ER: Emotion recognition	AT: Altertness traing
	1 day per week	PT: Perspective taking	WM: Working memory
**(P)**	Phonecall with therapist	SP: Social problem solving	SH: Shopping
